# Reinforced Nacre‐Like MXene/Sodium Alginate Composite Films for Bioinspired Actuators Driven by Moisture and Sunlight

**DOI:** 10.1002/smll.202406832

**Published:** 2024-10-06

**Authors:** Linchao Sun, Lixuan Che, Ming Li, Kai Chen, Xu Leng, Yaojia Long, Xiaoxi Guo, Matteo Palma, Yao Lu

**Affiliations:** ^1^ Department of Chemistry School of Physical and Chemical Sciences Queen Mary University of London London E1 4NS UK; ^2^ State Key Laboratory of Structural Analysis Optimization and CAE Software for Industrial Equipment Department of Engineering Mechanics School of Mechanics and Aerospace Engineering Dalian University of Technology Dalian 116024 P. R. China

**Keywords:** intelligent devices, nacre‐like structure, natural renewable energy, soft actuator

## Abstract

MXene‐based soft actuators have attracted increasing attention and shown competitive performance in various intelligent devices such as supercapacitors, bionic robots and artificial muscles. However, the development of robust MXene‐based actuators with multi‐stimuli responsiveness remains challenging. In this study, a nacre‐like structure soft actuator based on MXene and sodium alginate (SA) composite films is prepared using a straightforward solvent casting self‐assembly method, which not only enhances the mechanical performance (tensile strength of 72 MPa) but also diversifies the stimuli responsiveness of the material. The composite actuators can be powered by external stimuli from renewable energy sources, from moisture inducing a maximum bending angle of 190 degrees at a relative humidity (RH) of 91%, and sunlight irradiation generating a maximum curvature of 1.45 cm^−1^ under 100 mW cm^−2^. The feasibility of practical applications, including moisture‐responsive flowers and walkers, sunlight‐responsive oscillators, and smart switches, is demonstrated through comprehensive experimental characterization and performance evaluation. The work presented here provides insight into the design of robust actuators via the utilization and conversion of environmentally renewable energy sources.

## Graphical abstract

A soft actuator with nacre‐like structures of MXene and sodium alginate composite films is developed using a straightforward solvent casting self‐assembly method. This design enhances the mechanical performance and diversifies the stimuli responsiveness of the composite actuating material, thereby enabling its potential for various applications such as moisture‐responsive flower and walker, sunlight‐responsive oscillator and smart switch.

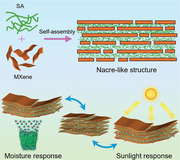

## 1 Introduction

In recent decades, the utilization and conversion of environmentally renewable energy sources have garnered significant attention in various fields. Soft actuators can capture external renewable energy (e.g., humidity,^[^ ^1^, ^2^, ^3^, ^4^ ^]^ sunlight,^[^ ^5^, ^6^ ^]^ heat,^[^ ^7^, ^8^ ^]^ magnetic fields^[^ ^9^, ^10^, ^11^ ^]^) and convert it into mechanical motions for specialized manipulations, granting them considerable potential applications in soft robots,^[^ ^12^, ^13^ ^]^ wearable devices,^[^ ^14^ ^]^ artificial muscles,^[^ ^15^ ^]^ energy generation, and conversion.^[^ ^16^ ^]^ Soft actuators have flexible deformation capabilities and soft contact with delicate objects. However, the mechanical performance is usually ignored when assembling soft actuators, limiting their functionality, durability and load‐bearing capacity. Therefore, it is highly desirable to develop methods for reinforcing soft actuating materials with high‐performance stimuli responsiveness.

As a novel 2D material that is widely applied in the field of soft actuators, MXene materials are a group of 2D transition metal carbides, nitrides and carbonitrides, where “M” denotes a transition metal (e.g., Ti, Cr, Mo), “X” represents carbon or nitrogen, and “ene” with the general formula of T_x_ indicates terminal functional groups (─O, ─F or ─OH). MXenes are typically synthesized by selectively etching the A‐group layers (e.g., Al, Si) from the MAX (M_n+1_AX_n_) phase precursors. Notably, titanium carbide (Ti_3_C_2_T_x_), a representative MXene, exhibits excellent hydrophilicity, photothermal conversion efficiency, electrical conductivity, and customizable layer spacing by grafting molecular chains,^[^ ^5^, ^17^, ^18^, ^19^ ^]^ giving them inherent advantages in the realm of actuator development. However, MXene nanosheets are not stable in an ambient environment because of oxidation,^[^ ^20^, ^21^ ^]^ that can weaken their super‐hydrophilicity and conductivity. Additionally, the mechanical tensile strength is an important index for evaluating the durability and stability of MXene‐based actuators. Therefore, pure MXene films offer no advantage in actuation and mechanical performance, especially due to their susceptibility to oxidation, which further reduces their service life. In this regard, the development of composite materials and the construction of reinforcement structures can allow the enhancement of mechanical performance.

Herein, a nacre‐like MXene/sodium alginate (MXSA) composite actuator was prepared using a straightforward water evaporation self‐assembly method. The integration of MXene and SA materials allowed us to exploit their complementary functions, enhancing the mechanical strength of the composite and diversifying actuating performance. This further facilitated multifunctionality and reconfigurability. Owing to the high hygroscopicity of SA and outstanding photothermal conversion efficiency of MXene, the MXSA composite films developed here exhibited multi‐stimulus‐responsiveness to external renewable stimuli including moisture and sunlight. Importantly, the nacre‐like structure markedly reinforced the mechanical performance of the MXSA composite actuator, yielding a tensile strength four times greater than that of pure MXene films. In addition, the feasibility of practical applications, such as moisture‐responsive flower, robotic arm and walker, sunlight‐responsive oscillator, and smart switch, were demonstrated. These applications are powered by natural renewable energy and possess adaptability to varying humidity and light intensities, providing valuable insight in the design of materials for energy conservation.

## 2 Results and Discussions

**Figure 1:**
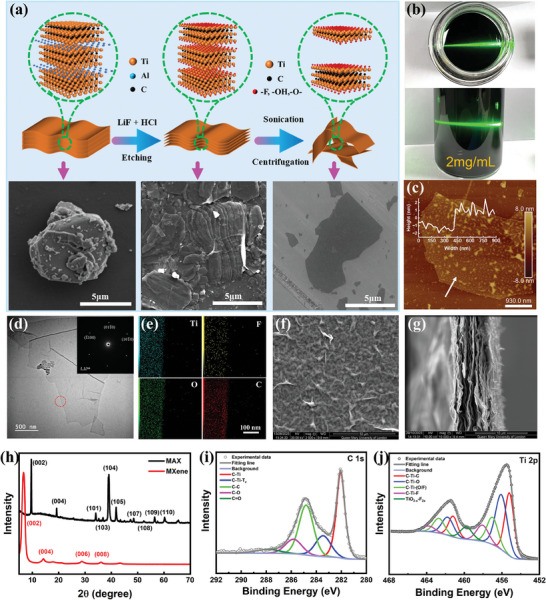
Synthesis and characterization of MXene nanosheets. a) Elaborate preparation, structure and surface morphology of MAX phase, intermediate, and MXene materials. b) Photograph of MXene dispersion with Tyndall effect. c) Topographical image and corresponding height profile of MXene nanosheets cast on Si substrate. d) TEM image and SAED pattern of MXene nanosheets. e) Corresponding EDS mappings of MXene nanosheets. f) Surface SEM image of MXene film. g) Cross‐sectional SEM image of MXene film. h) XRD pattern of MAX phase and MXene nanosheets. i) Deconvolution of C 1s peak of MXene. j) Deconvolution of Ti 2p peak of MXene.

MXene (Ti_3_C_2_T_x_) dispersion with monolayer and few layers was prepared by a typical exfoliating method. **Figure** **1**a shows the schematic diagram of the preparation process, the internal atomic structure and the corresponding scanning electron microscope (SEM) images of MAX phase, intermediate and MXene materials, respectively. Initially, the MAX phase presents a block structure. After hydrofluoric acid etching, the intermediate presents the hierarchical structure due to the delimitation of the Al layer. Subsequent sonication treatment induces the hierarchical structure of the intermediate, resulting in layers separation and yielding the MXene nanosheets, as monolayer or few layers. As shown in Figure 1b, it can be observed that the MXene dispersion in water presented a distinct Tyndall effect – scattering of a light beam by colloidal particles or particles in a transparent medium^[^ ^22^ ^]^ – when the laser beam passed through it, demonstrating the formation of colloid and successful synthesis of MXene nanosheets. SEM imaging (Figure S1, Supporting Information) reveals the surface morphology of MAX phased and MXene nanosheets, while the corresponding energy dispersive X‐ray spectroscopy (EDS) elemental mapping (Figure S2, Supporting Information) confirms the elimination of the Al layer after etching. Atomic force microscope (AFM) imaging and the corresponding height profile of MXene on the Si substrate are displayed in Figure 1c, respectively. MXene nanosheets exhibit a similar surface morphology to what was observed via SEM imaging. Additionally, the measurement from the height profile indicates that the thickness of the MXene nanosheets ranges from ≈1.5 to 3.5 nm, implying the successful preparation of monolayer and few layers MXene nanosheets.^[^ ^23^, ^24^, ^25^ ^]^ Transmission electron microscope (TEM, Figure 1d) and high‐resolution TEM imaging (Figure S3, Supporting Information) of MXene nanosheets show the typical characteristics of 2D materials, featuring a thin and transparent sheet with a large size. Additionally, the corresponding selected area electron diffraction (SAED) pattern (inset of Figure 1d) confirms the hexagonal structure and single crystallinity of the MXene.^[^ ^26^, ^27^ ^]^ The elemental mappings in Figure 1e further demonstrate the presence of Ti, C, F, and O elements and the successful introduction of terminated functional groups such as ─O, ─F, and ─OH in MXene nanosheets.

Typically, the pure MXene film was prepared by vacuum filtration method. Surface (Figure 1f) and cross‐sectional SEM imaging (Figures 1g and S4, Supporting Information) were conducted to observe the morphology of both the surface and interior structure of the MXene film. The stacking of MXene nanosheets took place at the interface in a highly ordered manner during the vacuum filtration process, resulting in a well‐aligned, smooth, and compact surface. The loosely lamellar structure has a higher specific surface area, facilitating the absorption and desorption of water molecules. This is the primary working mechanism that the MXene‐based materials can be developed for humidity‐ and light‐responsive actuators.

Figure 1h exhibits the X‐ray diffraction (XRD) patterns of the original MAX phase and MXene nanosheets. The characteristic diffraction peak corresponding to the (002) crystal plane of the MAX phase occurred at 9.6° and the intense (002) diffraction peak was broadened and shifted to 6.7° after etching, suggesting that MXene was successfully exfoliated. The other characteristic peaks of the MAX phase disappeared, indicating the complete elimination of the Al layer in prepared MXene.^[^ ^28^, ^29^, ^30^ ^]^ According to Bragg's Equation (2dsinθ = nλ),^[^ ^5^, ^31^ ^]^ the d‐spacing was calculated as 1.32 nm, which is consistent with the result of AFM test and theoretical thickness of MXene nanosheets with monolayer or few layers. X‐ray photoelectron spectroscopy (XPS) survey spectrum exhibits the characteristic peaks of Ti, C, O, and F elements. Specifically, as shown in Figure S5 (Supporting Information), the peaks corresponding to F 1s, Ti 2s, O 1s, Ti 2p, and C 1s are observed at binding energy values of 685, 564, 531, 456, and 284 eV, respectively.^[^ ^32^, ^33^, ^34^ ^]^ A negligible peak observed at ≈78 eV indicates the presence of a small amount of Al, likely originating from the derivatives after exfoliation.^[^ ^35^ ^]^ Figure 1i shows the fine‐fitting curve of the C 1s spectrum in MXene film. The peaks at binding energy values of 282.02, 283.43, 284.80, 285.85, and 287.78 eV correspond to C─Ti, C─Ti─T_x_, C─C, C─O and C═O bonds in sequence.^[^ ^36^ ^]^ The doublets in the fine splitting of Ti 2p (Figure 1j) are observed at 455.1 and 461.1 eV, 456.0 and 461.8 eV, 457.0 and 462.7 eV, 458.0 and 463.7 eV, 459.8 eV, which are assigned to C─Ti─C, C─Ti─O, C─Ti─(O/F), C─Ti─F and TiO_2‐x_─F_2x_, respectively.^[^ ^37^, ^38^, ^39^, ^40^, ^41^, ^42^ ^]^ The C─Ti─C component originates from the interior regions of the MXene layers.^[^ ^43^ ^]^ A variety of C─Ti─T_x_ bonds, such as C─Ti─O, C─Ti─(O/F) and C─Ti─F, are introduced during the exfoliating preparation process.^[^ ^30^, ^44^ ^]^ Additionally, TiO_2‐x_─F_2x_ bonds are generated as a result of oxidation.^[^ ^45^ ^]^ The XPS analysis provides substantial evidence for the formation of numerous termination functional groups on the MXene surface.

Flat MXSA composite films were prepared by a colloidal dispersion and a subsequent solvent casting self‐assembly method, as schematically shown in Figure S6 (Supporting Information). The resulting MXSA composite films, distinguished by varying MXene contents ranging from low to high, are sequentially designated as MXSA 0–6 (Figure S7, Supporting Information). Fourier transform infrared (FTIR) spectra (Figure S8, Supporting Information) verified the presence of functional groups in MXene and MXSA composite films, such as O─H, C─F and Ti─O bonds. Raman spectroscopy of MAX phase, MXene and MXSA films (Figure S9, Supporting Information) was employed to investigate the composition information and identify the chemical state of the MXSA composite. Figure S10 (Supporting Information) shows the XRD patterns for MXSA films 1–6. With the increase in the proportion of MXene in MXSA films, the slight blueshift of the characteristic peak (002) suggests a contraction of interlayer spacing within MXene nanosheets. This phenomenon is attributed to the diminished insertion of SA molecules and a reduction in disorder between MXene nanosheets.^[^ ^46^, ^47^ ^]^ Besides, the broad peaks at around 13.5° indicate an amorphous structure of SA in MXSA films,^[^ ^48^ ^]^ exhibiting a notable decrease in intensity as the SA content decreases. Notably, the XRD pattern shows no prominent peaks corresponding to oxidation products after a storage period of 3 months. This may be due to the sodium alginate film wrapping around the surface of MXene, which effectively obstructed oxygen contact and consequently slowed down the oxidation rate of MXene in MXSA composite films. The surface SEM images (Figure S11, Supporting Information) of MXSA films reveal that the surface morphology becomes smoother with the progressive incorporation of MXene nanosheets, gradually resembling to that of the pure MXene film, as shown in Figure S11a–h (Supporting Information). In addition, the corresponding EDS elemental mapping images in Figure S11i (Supporting Information) confirm the presence and uniform distribution of main elements including Ti, C, O, F, and Na on the MXSA film surface, where the high‐density carbon element in the background comes from carbon adhesive substrate.

Additionally, the cross‐sectional SEM images (Figure S12, Supporting Information) reveal a nacre‐like arrangement within the MXSA composite films, resembling the structural characteristics of reinforced “brick‐mortar”.^[^ ^49^ ^]^ The introduction of SA plays the role of adhesive to connect the MXene nanosheets, thereby the mechanical performance of MXSA composite film might be enhanced (see below). In addition, the high hydrophilicity and hygroscopicity of SA materials facilitate the moisture‐driven actuating performance of MXSA composite film due to a synergistic effect. Figure S12i (Supporting Information) exhibits the cross‐sectional EDS elemental analysis of the MXSA 6 film. These elemental mapping images further confirm the presence of main elements including Ti, C, O, F, and Na. Figure S13 (Supporting Information) shows the measured thickness of MXSA 0–6 films.

**Figure 2:**
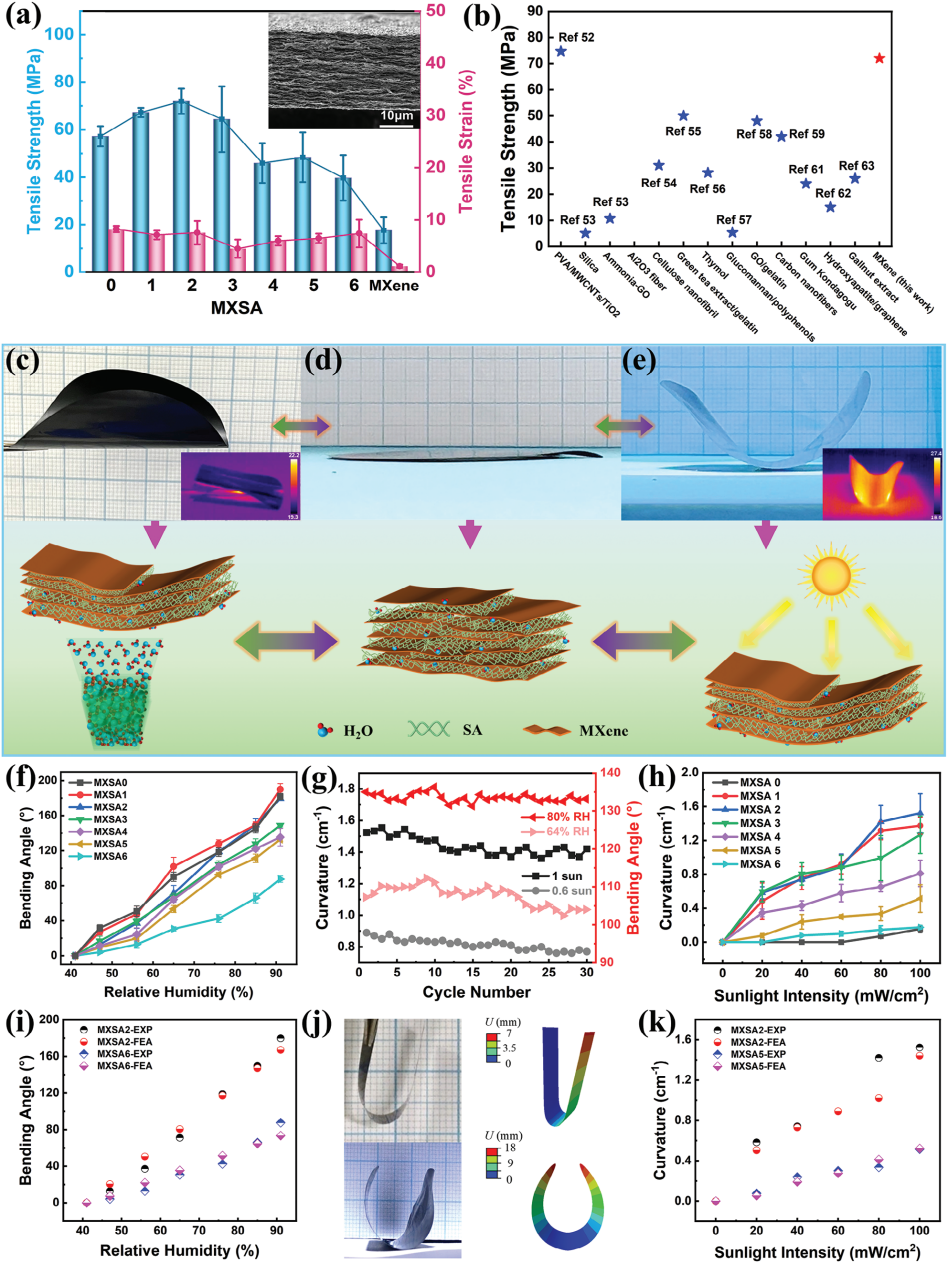
a) Mechanical performance of MXSA actuators with different proportions. b) Comparison of tensile strength of SA‐based composite films with different modifiers. c) Photograph of humidity‐induced bending and schematic of the humidity actuation mechanism of MXSA actuator. d) Photograph of MXSA actuator in its initial flat state. e) Photograph of sunlight‐induced bending and schematic of sunlight actuation mechanism of MXSA actuator. f) Bending angle of MXSA actuators as a function of relative humidity level. g) Stability of MXSA actuators under humidity and sunlight stimulation. h) Curvature of MXSA actuators as a function of sunlight intensity. i) Experimental (EXP) and simulated (FEA) bending angles of MXSA actuators under moisture stimuli. j) Comparison of experimental and simulation results. k) Experimental (EXP) and simulated (FEA) curvatures of MXSA actuators under sunlight exposure.

A mechanical test was conducted to validate the robustness of MXSA composite films, as shown in **Figure** **2**a. The typical tensile stress‐strain curve of MXSA films and MXene film (Figure S14, Supporting Information) demonstrated the enhancement of mechanical properties because of the adhesive effect of SA. In comparison to the MXSA 0 sample with a tensile strength of 57 MPa and the pure MXene film with a tensile strength of 17.6 MPa, when the content of MXene was low in MXSA composite films, the tensile strength had a significant increment, fluctuating within a range of 64–72 MPa. The MXSA 2 film with a thickness of 18 µm had the highest tensile strength of 72 MPa. The enhancement in tensile strength can be ascribed to the nacre‐like arrangement within the MXSA composite film, analogous to the structural characteristics of reinforced “brick‐mortar”.^[^ ^49^, ^50^, ^51^ ^]^ Here, MXene nanosheets act as the “bricks,” while SA functions as the “mortar”, to form a reinforced system that significantly improves the overall tensile strength of the MXSA composite films. However, with the further increase of MXene content in MXSA 3–6 composite, the tensile strength decreased dramatically to ≈50 MPa. The decline in tensile strength is mainly caused by the dominance of MXene with inherently poor mechanical properties owing to the weak interactions between MXene nanosheets. Additionally, the low tensile strain observed in pure MXene films is attributed to the weak interactions between layers, whereas pure SA films exhibit high tensile strain due to their flexible chain structure. With the increase of MXene content in MXSA films, the tensile strain presents a trend of initial decline followed by a subsequent rise, which is attributed to the toughness of SA at low MXene concentrations and the slippage of MXene sheets at higher MXene concentrations. Compared with other SA‐based composite films with different modifiers,^[^ ^52^, ^53^, ^54^, ^55^, ^56^, ^57^, ^58^, ^59^, ^60^, ^61^, ^62^, ^63^ ^]^ the MXSA composite films demonstrated significantly superior tensile strength in this work owing to the nacre‐like arrangement (Figure 2b).

The humidity‐responsive performance of MXSA actuators was confirmed by the fingertip approaching test. The snapshots of the reversible bending behavior of the circle‐shaped MXSA actuator triggered by fingertip are shown in Figure S15 and Video S1 (Supporting Information). When the fingertip approached the circular MXSA actuator and remained untouched, the MXSA actuator swung with the approaching and withdrawal of the fingertip. The swinging behavior was caused by the fluctuations in moisture around the fingertip. The relative humidity level near the finger position was higher than that of the ambient atmosphere, inducing a higher level of expansion of the MXSA film surface and leading to the bending in the opposite direction toward the fingertip. Simultaneously, the fingertip temperature higher than the ambient temperature played a synergistic effect to further enhance film deformation.

To confirm whether temperature or humidity from fingers is the decisive condition in film deformation, a hand interaction experiment was performed to observe the degree of deformation. As shown in Figure S16a (Supporting Information), the circular MXSA film exhibited an initial flat configuration. When placed on the palm without gloves, the circular MXSA film exhibited a noticeable bending deformation (Figure S16b, Supporting Information). However, there is no consistent phenomenon when the gloved palm was approached, thereby verifying that the bending of MXSA film was mainly triggered by the moisture rather than the temperature from the palm, as shown in Figure S16c (Supporting Information). The predominant responsiveness to moisture can also elucidate the occurrence of upward curvature under sunlight exposure.

The inherent hygroscopicity of SA and the hydrophilicity of MXene endow MXSA composite films with the capability of moisture‐responsive actuation (Figure S17, Supporting Information). Furthermore, the photothermal conversion of MXene makes the composite films sensitive to external light sources. In this regard, we investigated and analyzed the stimuli‐responsive performance and actuating mechanism of MXSA composite actuators (Figure 2c–e). The reversible bending behavior of the circle‐shaped MXSA actuator (Figure 2c,d) was observed to confirm its sensitivity to humidity. This responsiveness stems from the mechanism wherein water molecules are absorbed and desorbed. The MXSA actuator maintained its initial configuration, with consistent exposure to humidity at each point (ambient humidity 41% RH). Upon subjecting specific locations to elevated humidity levels, the actuator film exhibited notable expansion attributable to water molecule adsorption, leading to non‐uniform deformation of the overall structure. Once the external humidity input was removed, the MXSA actuator reverted to its original shape. The weight change ratio of MXSA 2 composite film exhibits an approximately linear increase as the humidity level rises from ambient to elevated conditions (Figure S18a, Supporting Information). Consequently, the average weight change ratios of MXSA composite films per 1% RH increment were measured, as shown in Figure S18b (Supporting Information).

In addition to the humidity responsiveness, the photothermal conversion capability of MXene makes the MXSA‐based actuators sensitive to sunlight illumination (Figure 2d,e). The circle‐shaped MXSA actuator initiated an upward bend when exposed to sunlight irradiation with an intensity of 1 sun (100 mW cm^−2^). Following the cessation of sunlight illumination, the MXSA actuator subsequently reverted to its initial flat state. The mechanism underlying the sunlight‐responsive actuation of MXSA actuators involves the temperature increase induced by the photothermal effect (inserted IR image in Figure 2e), which prompts the desorption of water molecules in MXSA composite, consequently leading to volume shrinkage. Specifically, there is a temperature gradient along the thickness direction of the MXSA actuator under the radiation of sunlight, resulting in a higher dissociation rate of water molecules and a more conspicuous reduction in volume at the top surface. The mismatch in volumetric changes along the thickness direction induces an upward bending of the MXSA actuator. Conversely, the MXSA actuator can undergo a process of reabsorbing water and expanding back to its initial state due to the reversible nature of hydration and dehydration.

To investigate and quantify the stimuli‐responsive sensitivity of MXSA actuators, the bending angles were measured in relation to varying humidity levels, while curvatures were assessed under different intensities of sunlight illumination. Specifically, the bending angles and curvatures were defined as the included angle and the maximum curvature, respectively. As shown in Figure 2f, the bending angles of MXSA composite actuators (6 mm × 32 mm) exhibit a notable increase from 0 to a maximum of 190 degrees when the external humidity levels were systematically elevated from 41% to 91% RH. The MXSA 0–2 composite films exhibit a similar maximum bending angle of ≈185 degrees at 91% RH. Additionally, there is a notable attenuation in bending angles with the augmentation of MXene nanosheet content in MXSA actuators, which is attributed to the heightened demand for deformation force due to increased thickness.

Besides, the curvature of MXSA actuators (diameter of 45 mm) as a function of sunlight intensity was further investigated, as depicted in Figure 2h. The curvature exhibits a positive correlation with the increase in sunlight intensity (0–100 mW cm^−2^), attributing to the enhancement of the photothermal effect under higher‐intensity sunlight irradiation. However, the curvature of MXSA actuators with different component proportions shows a trend of initially increasing and then decreasing as the MXene content increases. The comparison of the curvature among different MXSA actuators under identical light intensity is shown in Figure S19 (Supporting Information). When the mass fraction of MXene nanosheets in MXSA actuators is low, the enhanced photothermal effect and heat accumulation resulting from the increase of MXene content facilitate the desorption of water molecules and further produce higher curvature bends. However, at higher mass fractions of MXene nanosheets, the deformation of MXSA actuators becomes more difficult due to thickness constraints, thereby further attenuating the bending of MXSA actuators. Figure S20 (Supporting Information) exhibits the corresponding real‐time curvature curve of the MXSA 2 actuator. The MXSA 2 actuator could bend with a maximum curvature of 1.45 cm^−1^ under 14 s of 100 mW cm^−2^ sunlight illumination. Subsequently, it gradually returned to its initial flat state in 155 s after being shielded from sunlight irradiation. Table S1 (Supporting Information) presents a comparison of stimuli responsiveness and tensile strength between the MXSA‐based actuating material and other actuating materials in the literature. Furthermore, the durability and stability of the MXSA actuator were evaluated by measuring curvatures under the stimulation of different humidity levels and sunlight intensities across multiple cycles. As shown in Figure 2g, the magnitude of the fluctuations in the extent of deformation remained within a reasonable range, indicating the exceptional stability of the MXSA actuators.

Finite element analysis (FEA, see Supporting Information) was conducted using the commercial package ABAQUS^[^ ^64^ ^]^ to study the bending deformation of the MXSA actuators when subjected to external moisture and sunlight stimulation. From the modeling, the moisture expansion coefficients of MXSA 2 and MXSA 6 were approximated to be 4.8% and 3.4%, respectively. The experimental (EXP) and simulated (FEA) bending angles of MXSA actuators under moisture stimulation (Figure 2i) confirm that the model matches the experimental results well. Similarly, the equivalent shrinkage coefficients of MXSA 2 and MXSA 5 were approximately determined to be 0.02% and 0.009%, respectively. The experimental (EXP) and simulated (FEA) curvatures of MXSA actuators under sunlight stimulation (Figure 2k) indicate a good agreement between the model and experimental results. Additionally, the comparison of experimental and simulated visualized deformation results (Figures 2j and S21 and 22, Supporting Information) further demonstrates that the finite element model accurately reflects the experimental observations.

**Figure 3:**
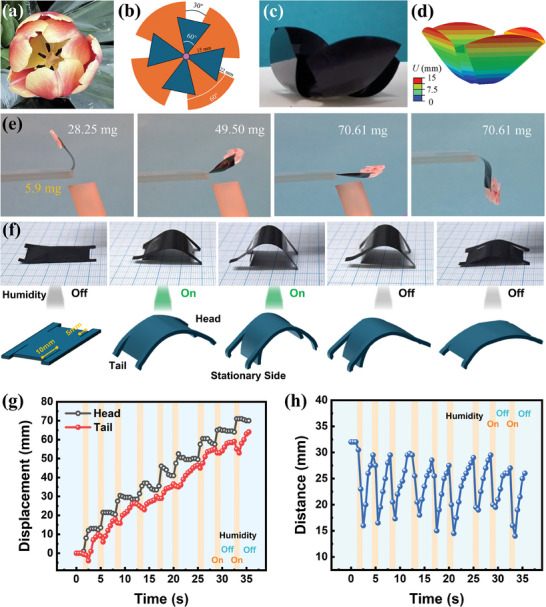
Humidity‐controlled‐MXSA actuators. a) Photographs of natural flower petals. b) Schematic diagram of biomimetic flower‐inspired MXSA actuator. c) Snapshot of open and close flower‐inspired MXSA actuator. d) FEA result of flower‐inspired MXSA actuator. e) Photographs of MXSA actuator for lifting different weights under 88.1% RH stimulation. f) Representative snapshots and corresponding schematics of biomimetic walker under moisture stimulation. g) Real‐time displacement of the head and tail with humidity on and off. h) Real‐time distance between head and tail of MXSA film with humidity on and off.

In nature, flower petals can open or close in response to light or temperature changes, as shown in **Figure** **3**a. Mimicking the biological behavior of flower petals in response to environmental stimuli, a humidity‐controlled flower‐inspired actuator (Figure 3b) was developed, achieving petal closure in high humidity conditions and petal opening when humidity levels decreased (Figure 3c; Video S2, Supporting Information). The deformation of a flower‐inspired actuator driven by humidity was simulated, as shown in Figures 3d and S23 (Supporting Information). The enhanced tensile strength significantly improved the load‐bearing capabilities of MXSA actuators, facilitating their application in weightlifting scenarios. As shown in Figure 3e, when exposed to humid air with a relative humidity of 88.1%, a rectangular MXSA actuator (5.9 mg) was able to bend and lift objects of varying weights (28.25, 49.50, and 70.61 mg). The maximum weight that can be lifted by the actuator was ≈12 times its own weight. Upon removal of the external humidity, the actuator reverted to its natural droop under the action of gravity.

Inspired by the directional motion of a natural inchworm – where the organism propels its body forward, securing its tail with claws and simultaneously extending its head forward – a biomimetic walker was engineered, integrating bendable components of different lengths. The representative snapshots and schematic diagrams of the walking principle of the biomimetic walker are illustrated in Figure 3f. The biomimetic walker maintained the flat stationary state in the initial stage, and subsequently bent upward upon the external humidity stimulation. Due to the constraints of the bendable head end with low length, the bending curvature of the tail end was larger than that of the head end, ensuring higher frictional force at the tail and recognizing it as a stationary side to prevent backward movement. After the removal of the external humidity, the induced lower bending led to the forward extension of the head end, generating continuous directional locomotion when alternately applied and removed by humidity stimulation (Video S3, Supporting Information). Figure 3g illustrates the real‐time displacement of both the head and tail under the manipulation of humidity. Following nine walking cycles, the displacement of the head of the biomimetic walker was ≈70 mm in 32.5 s. The real‐time distance fluctuation between the head and tail as a function of the time of the biomimetic walker is shown in Figure 3h.

**Figure 4:**
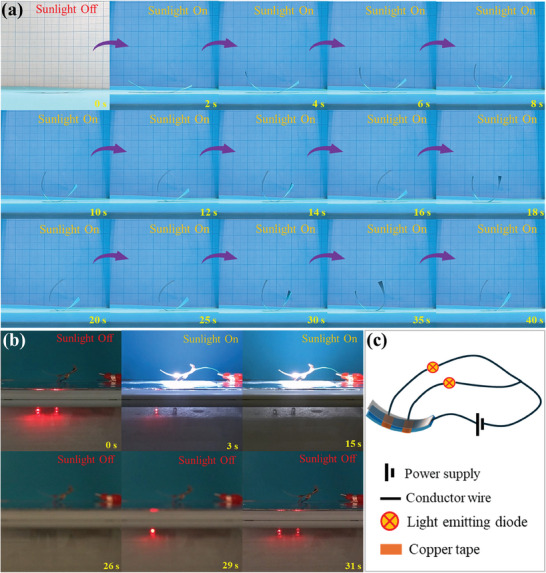
Sunlight‐controlled‐MXSA actuators. a) Self‐oscillating motion of MXSA composite actuator under constant sunlight illumination. b) Representative snapshots of LEDs sequentially turned on and off under sunlight control. c) Schematic diagram of the smart light‐controlled switch.

The sunlight‐responsive sensitivity accompanied with the superior conductivity of MXSA actuators makes them excellent candidates for applications in photo‐responsive devices. An example of a potential application for an MXSA actuator is an oscillator under the constant radiation of sunlight, as shown in **Figure** **4**a. Similar to the natural phenomenon of phototropism observed in sunflowers, the ribbon‐shaped MXSA actuator exhibits a tendency to bend toward the direction of the light source due to the generation of temperature gradient. In the initial stage of bending, the center of gravity of the MXSA actuator rose along the central axis, producing a stable upward bending motion. When the actuator bent to a certain curvature, the center of gravity shifted away from the central axis, attributed to the self‐shadowing on both sides. The continuous alternation of bending and self‐shadowing resulted in the generation of self‐sustained oscillations (Video S4, Supporting Information). Upon removal of the sunlight illumination, the oscillations stopped and the MXSA oscillator returned to its initial shape.

To visually display the conductivity of MXSA composite films, a closed circuit employing a strip of MXSA film as a conductor was set up, as shown in Figure S24a–f (Supporting Information). The conductive copper foil tape with a fixed distance (1.5 cm) ensures that the length of the MXSA film connected to the circuit is consistent. The conductivity is determined by the brightness of the circuit light emitting diode in the circuit. Due to the low content of MXene, the decisive component for conductivity, MXSA 1 and MXSA 2 couldn't light up the light‐emitting diode, suggesting the high resistance and low conductivity of these samples. The faint glow was first observed in the circuit connected with MXSA 3. The circuits assembled with MXSA 4–6 exhibit significantly enhanced luminescence and improved conductivity, which is attributed to the increase of MXene content in these samples. The variations in surface resistance of the MXSA composite film at varying distances were measured and shown in Figure S24g (Supporting Information). The results indicate that the surface resistance of MXSA composite film significantly decreases with increasing MXene content, while it slightly decreases with increasing distance between measurement points. Therefore, higher MXene content and shorter measuring distances are advantageous for the application of MXSA composite films in electronic switches.

Considering the conductivity and sunlight‐responsive sensitivity of our MXSA actuator, the MXSA 5 films were designed for a sunlight‐controlled smart switch with a graded response. The optical snapshots and schematic diagram of the circuit are depicted in Figure 4b,c, respectively. The terminals of a parallel circuit are fixed to two separate conductive copper strips at each end. The remaining terminals of the parallel circuit are unified and linked to the negative pole of the power supply. To ensure a secure attachment, one end of the ribbon‐shaped MXSA actuator was affixed onto a curved insulated base connected with the positive terminal of the power supply, while the other end remained unrestricted, allowing for free movement. When the sunlight was switched off, the circuit remained intact, and the two light‐emitting diodes (LEDs) were illuminated. When the sunlight was turned on, the MXSA actuator bent upward, moving away from the conductor, which resulted in the disconnection of the one circuit near the free end and the extinguishing of the LED. As the duration of exposure to sunlight prolonged, the degree of bending further increased. Consequently, the second circuit became open, leading to the deactivation of the second LED. Conversely, when the sunlight was turned off, the bent MXSA actuator returned to its original shape and the LEDs were lighted up again in sequence (Video S5, Supporting Information). The sunlight‐controlled switch system successfully achieved the function of a graded response, demonstrating its capability to be tailored for light‐intensity adaptive smart switching applications. In conditions of strong natural sunlight, all lights are deactivated, while during periods of weak natural sunlight, the number of lights to be illuminated is determined by the intensity of the sunlight, offering valuable insights for energy conservation.

## 3 Conclusion

In conclusion, we developed nacre‐like structure MXSA actuators, comprised of MXene (Ti_3_C_2_T_x_) and SA composite film; this was achieved via a straightforward solvent casting self‐assembly process. The incorporation of a nacre‐like structure enhanced the mechanical performance of MXSA films, resulting in a maximum tensile strength of 72 MPa. In addition, the composite actuators were shown to be sensitive to moisture and sunlight due to the high hygroscopicity of SA and the outstanding photothermal conversion efficiency of MXene nanosheets. A moisture and temperature gradient along the thickness direction led to the association/dissociation of water molecules, inducing unsymmetrical deformation of the MXSA actuators. The composite actuators exhibited unidirectional phototactic bending behavior, achieving a maximum curvature of 1.45 cm^−1^ under sunlight illumination of 100 mW cm^−2^. The potential applications, including a humidity‐responsive flower and walker, a sunlight‐responsive oscillator and a smart switch, were demonstrated through specific configuration design and external stimuli manipulation. Notably, these applications are powered by natural sunlight and possess adaptability to varying light intensities, providing valuable insights in the context of energy conservation.

## 4 Experimental Section

### Materials

Titanium aluminum carbide MAX phase powder (Ti_3_AlC_2_, 325 mesh, purity > 99%) was provided by Nanografi Nano Technology. Lithium fluoride (LiF, 300 mesh) and sodium alginate (SA) were purchased from Sigma–Aldrich Company Ltd. Hydrochloric acid (HCl, 37% solution in water) was purchased from Fisher Scientific UK Ltd. All chemicals were used without further purification.

### Preparation of MXene Dispersion

The delaminated MXene dispersion with monolayer and few layers was synthesized by the exfoliating method. The typical synthesis process is as follows: LiF powder (3.2 g) was added into the 40 mL 9 m HCl solution and stirred for 20 min to completely dissolve. Subsequently, 2 g of Ti_3_AlC_2_ powder (MAX phase) was then slowly added into the mixed solution. The etching process was carried out at 40 °C for 48 h with a stirring rate of 400 rpm to remove the Al layer in Ti_3_AlC_2_ powder. The delaminated MXene was washed successively with 2 m HCl solution and deionized water and centrifuged repeatedly at 5000 rpm for 5 min until the pH of the supernatant reached ≈6. After that, the precipitate was re‐dispersed in 40 mL of deionized water and subjected to sonication in an ice bath at 800 W for 30 min under the protection of Ar. Following the replenishment of ice, the sonication of the dispersion continued at 800 W for an additional 30 min. The Ti_3_C_2_T_x_ colloidal solution was obtained through centrifugation at 3500 rpm for 30 min. The supernatant containing single‐layer MXene nanoflakes was collected by separating the precipitation. The concentration of Ti_3_C_2_T_x_ dispersion was determined by weighting the MXene film obtained by vacuum filtration, which was measured at ≈10 mg mL^−1^.

### Preparation of MXene Dispersion

The MXSA composite films in this work were prepared through a facile colloidal dispersion and subsequently solvent casting method. First, SA powders, comprising 2 wt.% mass fractions, were dissolved in deionized water and underwent continuous mechanical stirring at 70 °C for 1 h, resulting in the formation of a colloidally homogeneous SA solution. An equal quantity of the 2 wt.% SA solution, amounting to 2.5 g, was introduced into six identical vials. The different volumes (0.05, 0.25, 0.5, 1.5, 2.5, and 5 mL) of the obtained MXene dispersion (10 mg mL^−1^) were added to six vials containing SA solution. After 8 h of mechanical stirring, a homogeneous mixed mother liquor was obtained and subsequently poured into six Petri dishes (diameter of 50 mm, height of 12 mm). The uniform and desiccative MXSA composite films were formed and peeled off from the Petri dishes substrate, followed by the natural evaporation in a chamber with constant temperature and humidity (20 °C, 39% RH) for 48 h. These MXSA films, characterized by varying MXene contents from low to high, are sequentially labeled as MXSA 1–6. The pure SA film without MXene modification prepared under the same conditions is labeled as MXSA 0.

### Characterization Techniques

Transmission Electron Microscopy (TEM) images were taken by a JEOL F200 multi‐purpose electron microscope. The morphology and components of the as‐prepared films were characterized by scanning electron microscopy (SEM) with FEI Inspect F and energy‐dispersive X‐ray spectroscopy (EDS), respectively. The crystal structures of MXene materials were identified using X‐ray diffraction (XRD) analysis, which was collected by a PANalytical CubiX^3^ X‐ray diffractometer equipped with PIXcel 1D detector using Ni‐filtered Cu‐Kα radiation (λ = 1.5418 Å). The topography and thickness of MXene nanosheets were tested with a Bruker Dimension Icon atomic force microscope (AFM) operated in PeagFrorce Tapping mode via the use of Bruker ScanAsyst Air tips. The composition and chemical bonding were analyzed by X‐ray photoelectron spectroscopy (XPS, Thermo Fisher Scientific, K‐ALPHA) and the spectral fitting was implemented by Avantage software. Fourier transform infrared (FTIR) spectra were obtained using a PerkinElmer Spectrum 65 spectrometer within a scan range of 4000–600 cm^−1^, featuring a resolution of 1 cm^−1^. The temperature distributions on the film surface were recorded using an infrared thermal imaging camera (RS Pro IR Thermal Imager, RS‐9875). Raman spectroscopy was measured by Renishaw inVia Reflex Spectrometer System under 442 and 633 nm laser excitation in the range of 100–2000 cm^−1^. In addition, Stimulus‐responsive performance evaluations were conducted in a controlled laboratory environment with stable temperature and humidity levels, where the ambient temperature and relative humidity were ≈20 °C and 39%, respectively. The ambient temperature and humidity level in the chamber were recorded by a multifunctional moisture meter (RS Pro moisture meter, DT‐229). An AM 1.5G sunlight irradiance was excited by a solar simulator (SciSun‐150, SCIENCETECH), equipped with a 150 W xenon arc lamp, and the intensity of sunlight was calibrated by a silicon reference cell. The wettability of MXSA composite film surfaces was observed and analyzed using a Kruss DSA25S drop‐shape analyzer. The photographs and videos recording actuating motions were captured by a digital camera (Sony, DSC‐HX400V). The mechanical tests in this work were performed by an Instron 3342 universal testing machine. The composite films were prepared as rectangular specimens and tested at a loading rate of 1 mm min^−1^ for tensile tests until fracture.

## Conflict of Interest

The authors declare no conflict of interest.

## Supporting information



Supporting Information

Supplemental Video 1

Supplemental Video 2

Supplemental Video 3

Supplemental Video 4

Supplemental Video 5

## Data Availability

The data that support the findings of this study are available from the corresponding author upon reasonable request.
