# Supplementary material for: Reinforced Nacre‐Like MXene/Sodium Alginate Composite Films for Bioinspired Actuators Driven by Moisture and Sunlight
Source: Small. 2024 Oct 6;20(51):2406832. doi: 10.1002/smll.202406832 (PMC11657044; doi:10.1002/smll.202406832)
Supplement: Supplementary file 1 — Supporting Information [file SMLL-20-2406832-s003.docx]

**Reinforced nacre-like MXene/sodium alginate composite films for bioinspired actuators driven by moisture and sunlight**

*Linchao Sun, Lixuan Che, Ming Li, Kai Chen, Xu Leng, Yaojia Long, Xiaoxi Guo, Matteo Palma and Yao Lu**

L. Sun, K. Chen, X. Leng, Y. Long, Dr. X. Guo, Prof. M. Palma, Dr. Y. Lu

Department of Chemistry, School of Physical and Chemical Sciences, Queen Mary University of London, London E1 4NS, UK

L. Che, Prof. M. Li

State Key Laboratory of Structural Analysis Optimization and CAE Software for Industrial Equipment, Department of Engineering Mechanics, School of Mechanics and Aerospace Engineering, Dalian University of Technology, Dalian 116024, P. R. China

*Corresponding email: [yao.lu@qmul.ac.uk](mailto:yao.lu@qmul.ac.uk)

**
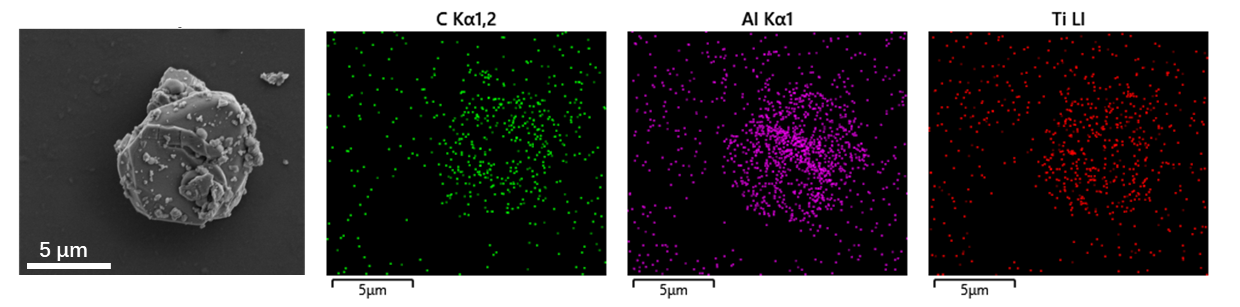
**

Figure S1 SEM image and EDS analysis of MAX phase.

In order to characterize the morphology and confirm the elimination of the Al layer after etching, SEM imaging and corresponding EDS elemental mapping were performed on both the MAX phase and the resultant MXene nanosheets. The SEM image (Figure S1) reveals the surface morphology of a block of MAX phase with a layer-structured configuration. The corresponding elemental mapping images indicate the uniform distribution of carbon (C), aluminium (Al), and titanium (Ti) across the entire MAX block, these being constituent elements of the MAX phase.

**
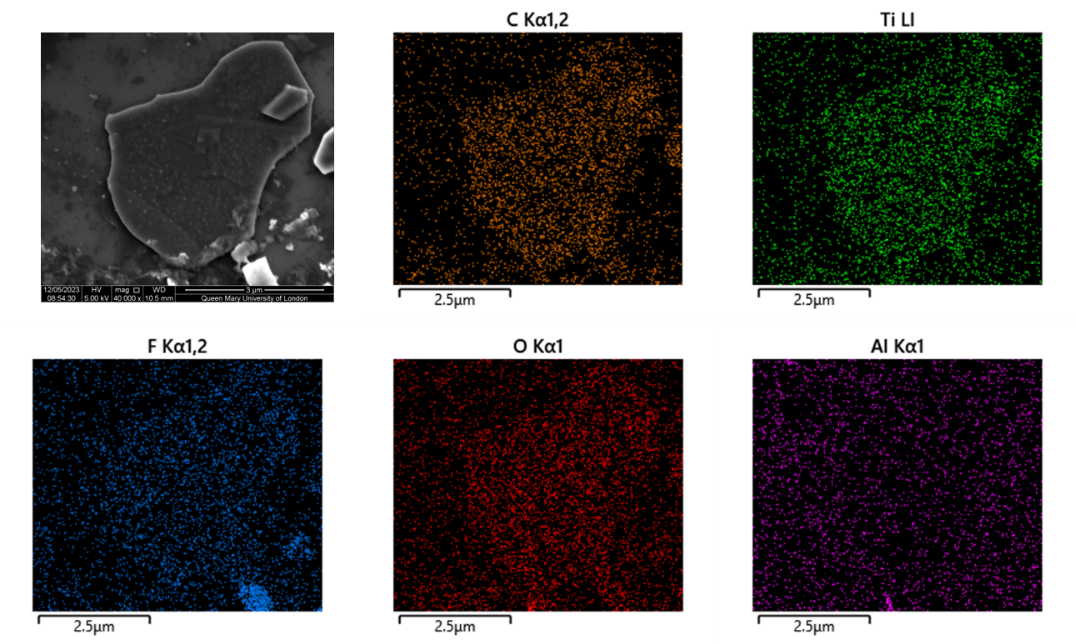
**

Figure S2 SEM image and EDS analysis of MXene nanosheets.

Figure S2 exhibits the SEM image and the corresponding EDS mapping analysis of the MXene nanosheets. The flake size and surface morphology of MXene nanosheets can be clearly observed in the SEM image. There are no obvious boundaries of MXene nanosheets in the Al elemental mapping image, suggesting that the Al layer was completely removed from the MAX phase during the hydrofluoric acid etching process. Simultaneously, the distinguishable boundaries of MXene in Ti, C, F and O elemental mapping images indicate the presence of these elements in MXene, attributing to the introduction of terminated functional groups such as ─O, ─F and ─OH.

**

**

Figure S3 High-resolution TEM image of MXene nanosheets.

**
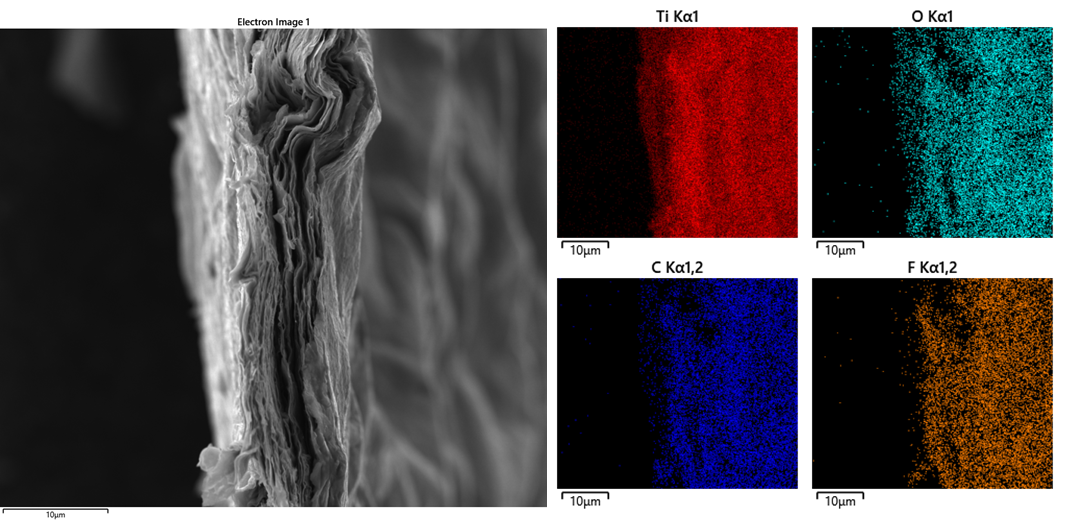
**

Figure S4 Cross-sectional SEM image and EDS analysis of MXene film.

To investigate the composition and confirm the elemental distribution of MXene film, the cross-sectional elemental mapping was implemented, as shown in Figure S4. Due to the successful removal of the Al layer, the elemental mappings confirm the presence of main elements such as Ti, C, O and F, where the O and F elements are attributed to the introduction of the terminated functional groups such as ─O, ─F or ─OH during the hydrofluoric acid etching process. There is no apparent local agglomeration of Ti, C, O and F elements in mappings throughout the film volume, The uniform distributions of these elements further demonstrate the well-ordered arrangement of MXene nanosheets when employing vacuum filtration.


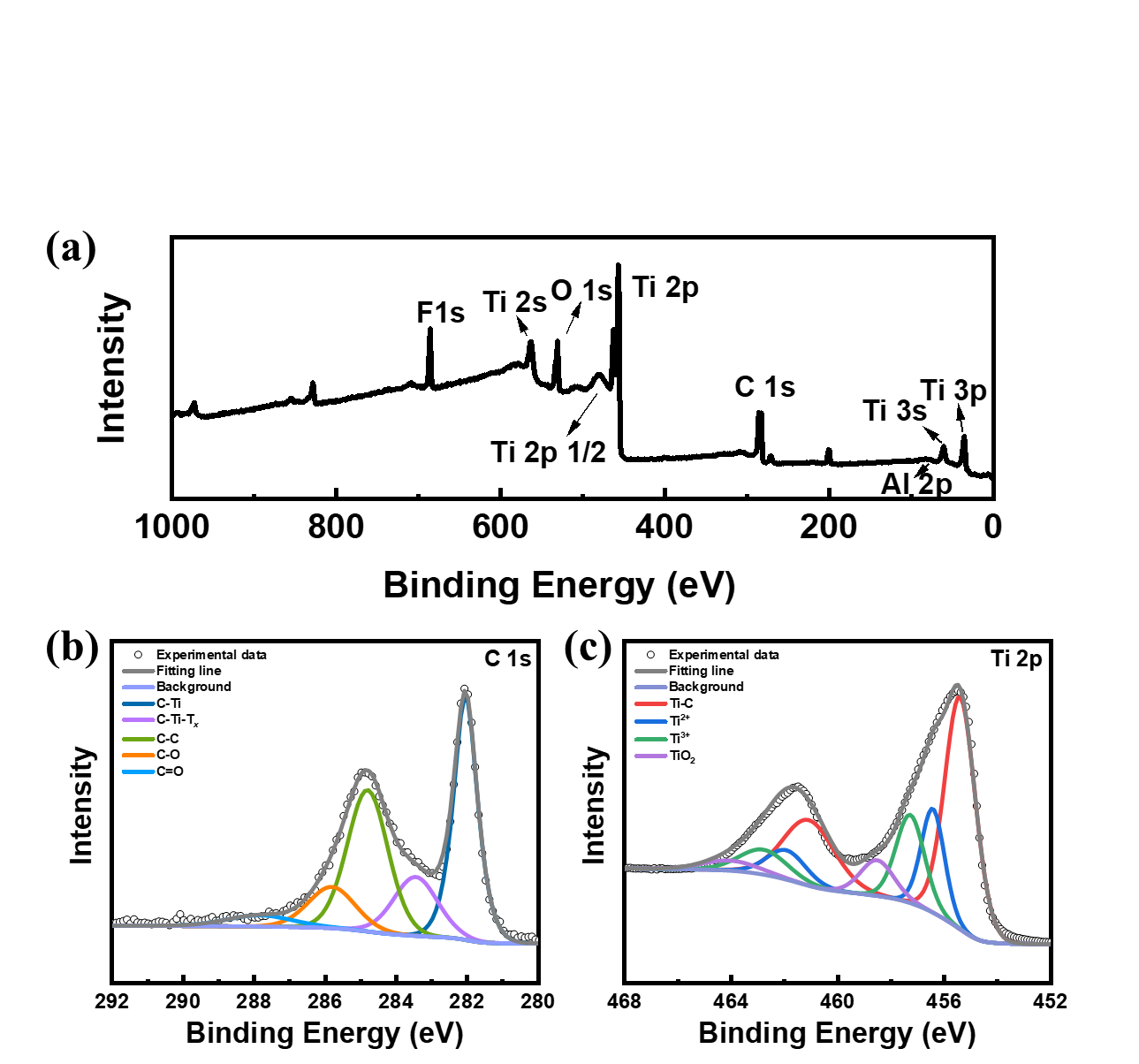


Figure S5 XPS full survey scan spectrum of MXene.


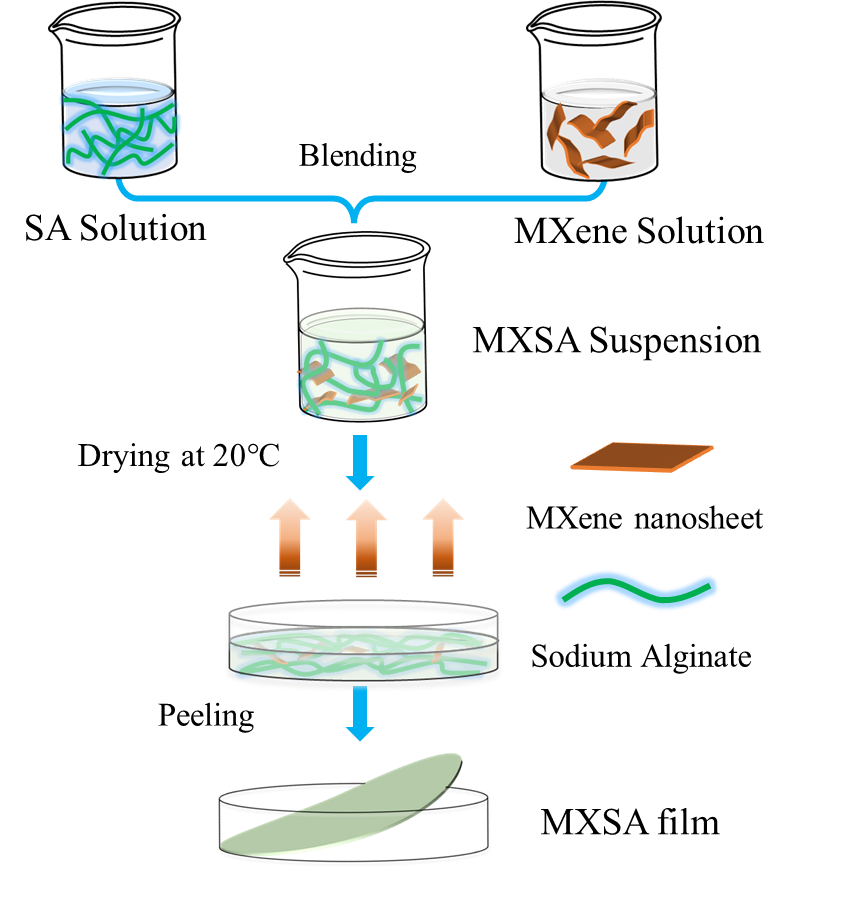


**Figure S6** Schematic of the preparation process of MXSA composite actuator.


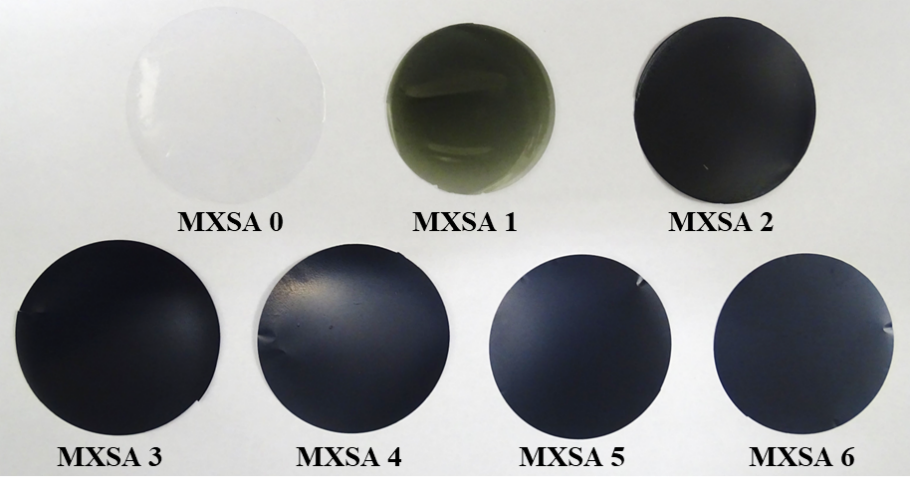


**Figure S7** Optical images of the obtained MXSA composite films.

Figure S8 FTIR spectra of pure MXene film and MXSA composite film.

The broad absorption peaks at 3485 cm^-1^ are attributed to the stretching vibration of O–H in the absorbed external water and strongly hydrogen-bonded –OH or extremely strong coordinated H_2_O due to the hydrophilicity of MXene.^[1]^ The band corresponding to the C–O stretching vibration at 1637cm^-1^ originated from the hydroxyl terminal functional groups.^[2]^ Besides, the peaks at 1101 cm^-1^ and 620 cm^-1^ appear in the FTIR spectrum of MXene, which are assigned to the vibrations of fluorine-terminated C–F and Ti–O bonds, respectively.^[3]^ Compared with pristine MXene, the FTIR spectrum of MXSA composite film presents a decrease in peak intensity due to the incorporation with SA, whereas the characteristic peaks can still be observed in the FTIR spectrum of MXene.


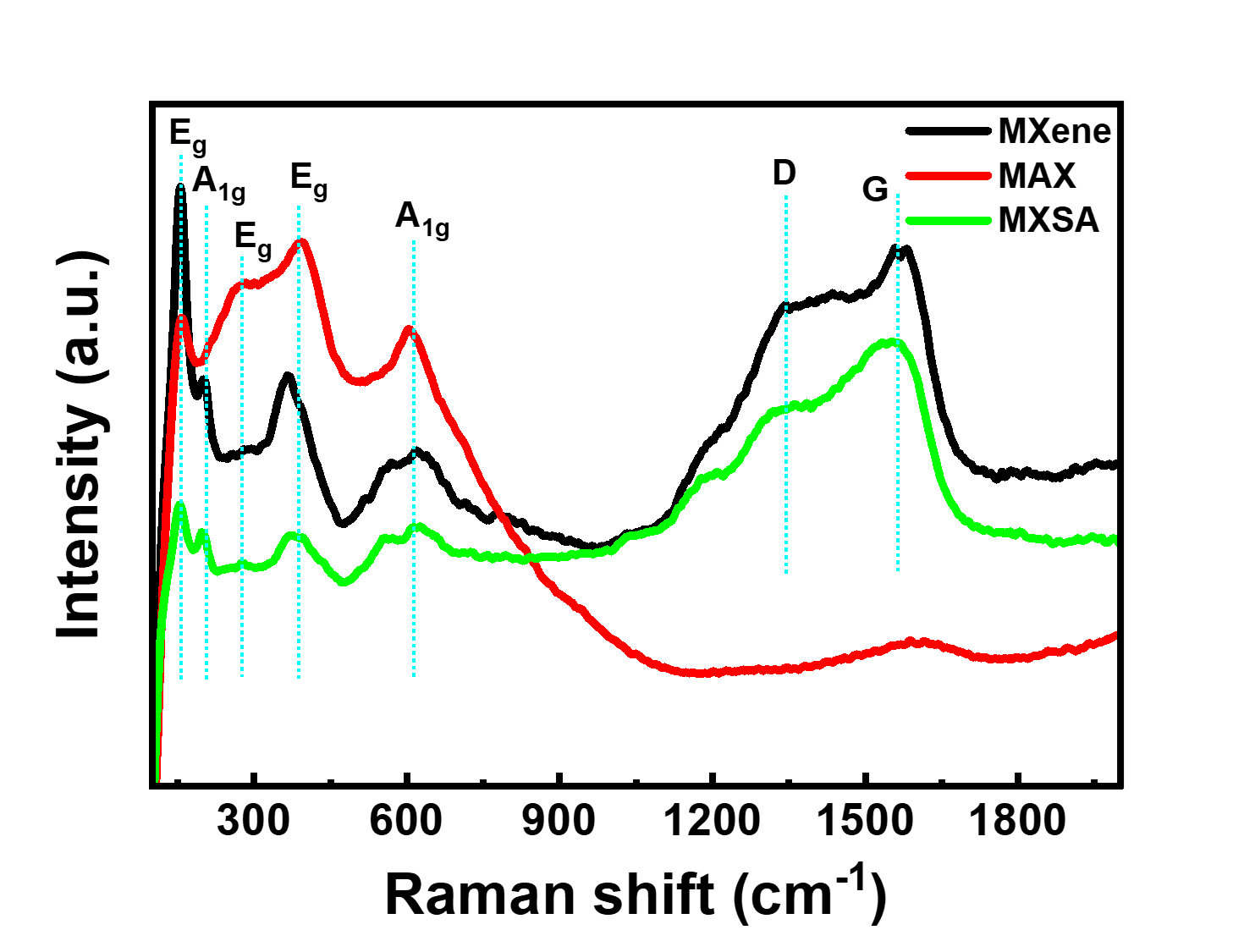


Figure S9 Raman spectra of MAX phase (excited at 442 nm), MXene nanosheets and MXSA composite film (excited at 663 nm).

The peaks at 159 and 207 cm^-1^ are associated with the in-plane and out-of-plane vibrations of Ti and C atoms of Ti–C (E_g_, and A_1g_ symmetries).^[4]^ The characteristic peaks at 273 and 394 cm^-1^ are assigned to the shear and longitudinal vibration of Ti and Al atoms in the MAX phase (E_g_ modes); their disappearance in the MXene spectrum confirms the removal of Al atoms after HF etching.^[5]^ The bands observed at 613 cm^-1^ are ascribed to the A_1g_ vibrational mode of C atoms of Ti–C.^[6]^ Furthermore, two additional peaks in the range of 1050-1800 cm^-1^ are assigned to the D band and G band of graphitic carbon, suggesting the presence of carbon and structural disorder in MXene.^[5, 7]^ The intensity of these two peaks in MXene is higher than that in the MAX phase, indicating an increased surface carbon exposure in MXene, which is beneficial to enhance the photothermal conversion efficiency.^[5, 8]^ The result of the Raman spectrum further demonstrates the effective elimination of Al layers in the MAX phase and the successful preparation of MXene nanosheets.


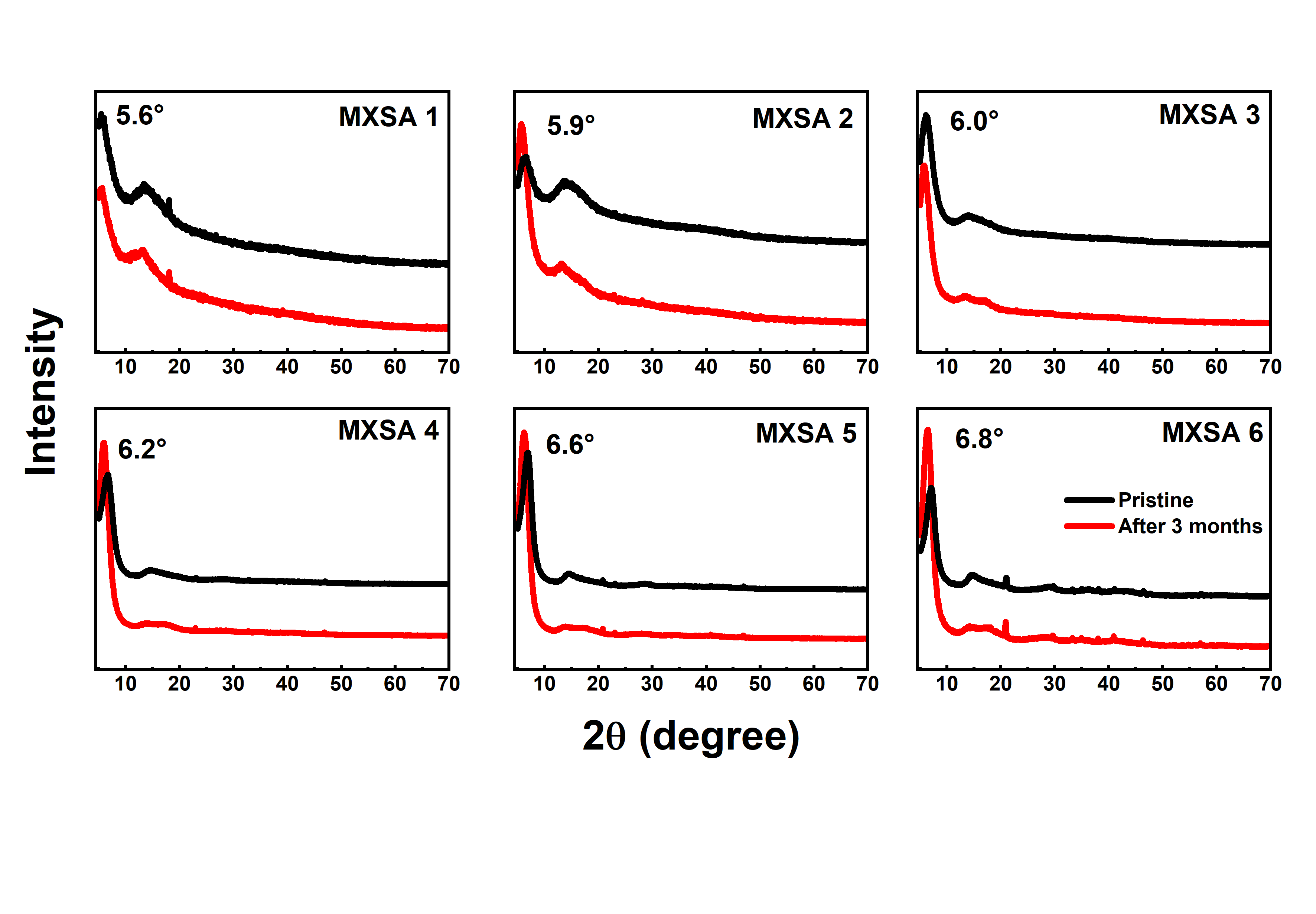


Figure S10 XRD patterns of MXSA composite films with different composition proportions.


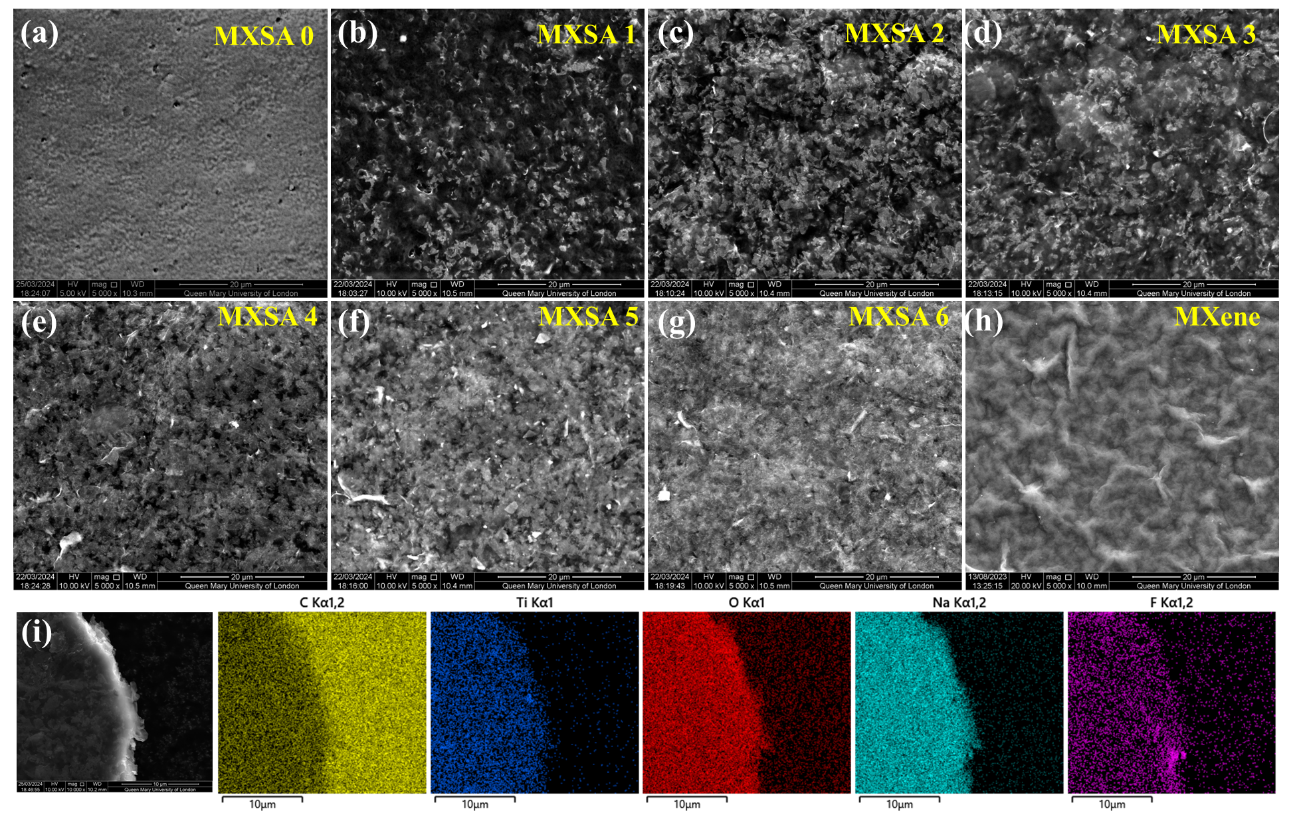


Figure S11 SEM images of (a-g) MXSA 0-6 film surface, (h) pure MXene film surface and (i) the corresponding EDS elemental maps of MXSA 4 film surface.


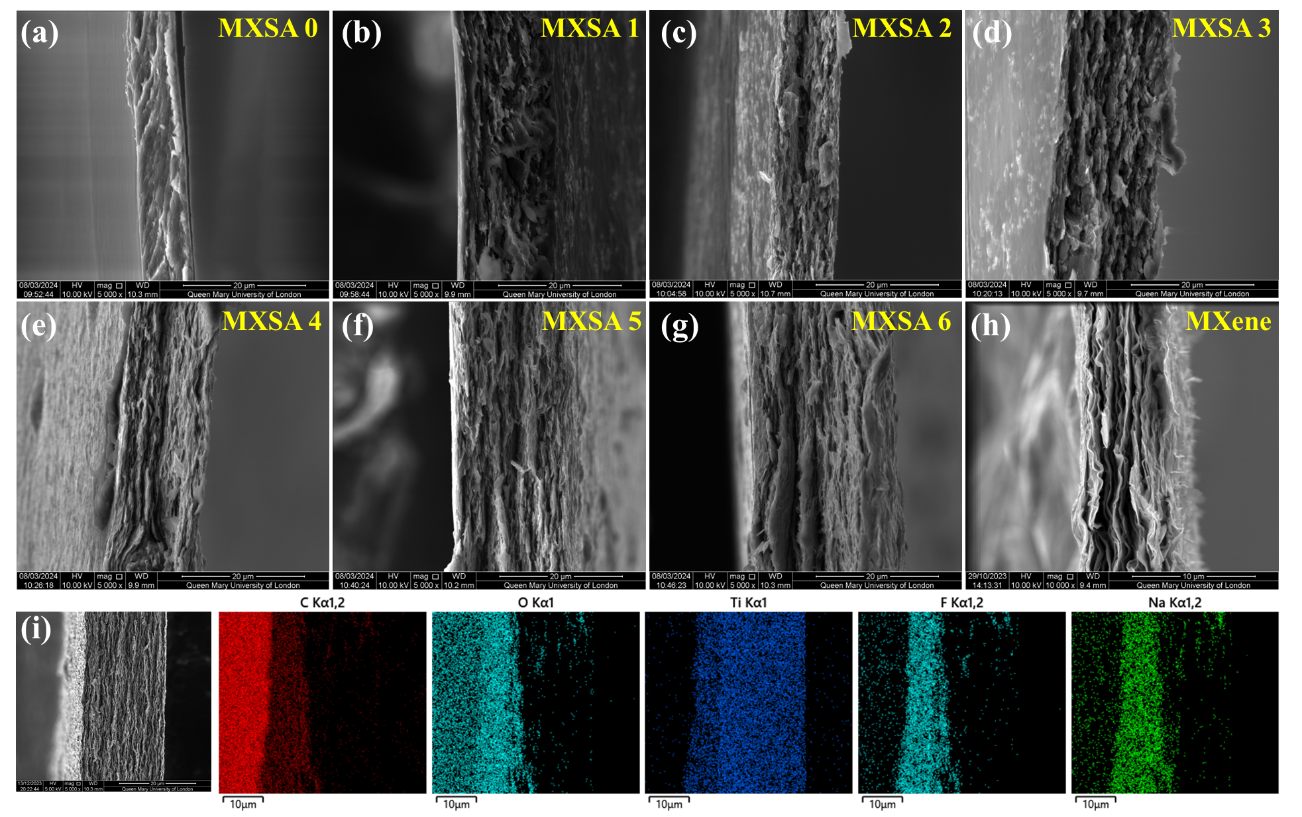


Figure S12 Cross-sectional SEM images of MXSA films with different composition proportions and the corresponding elemental mapping analysis of MXSA 6 film profile.


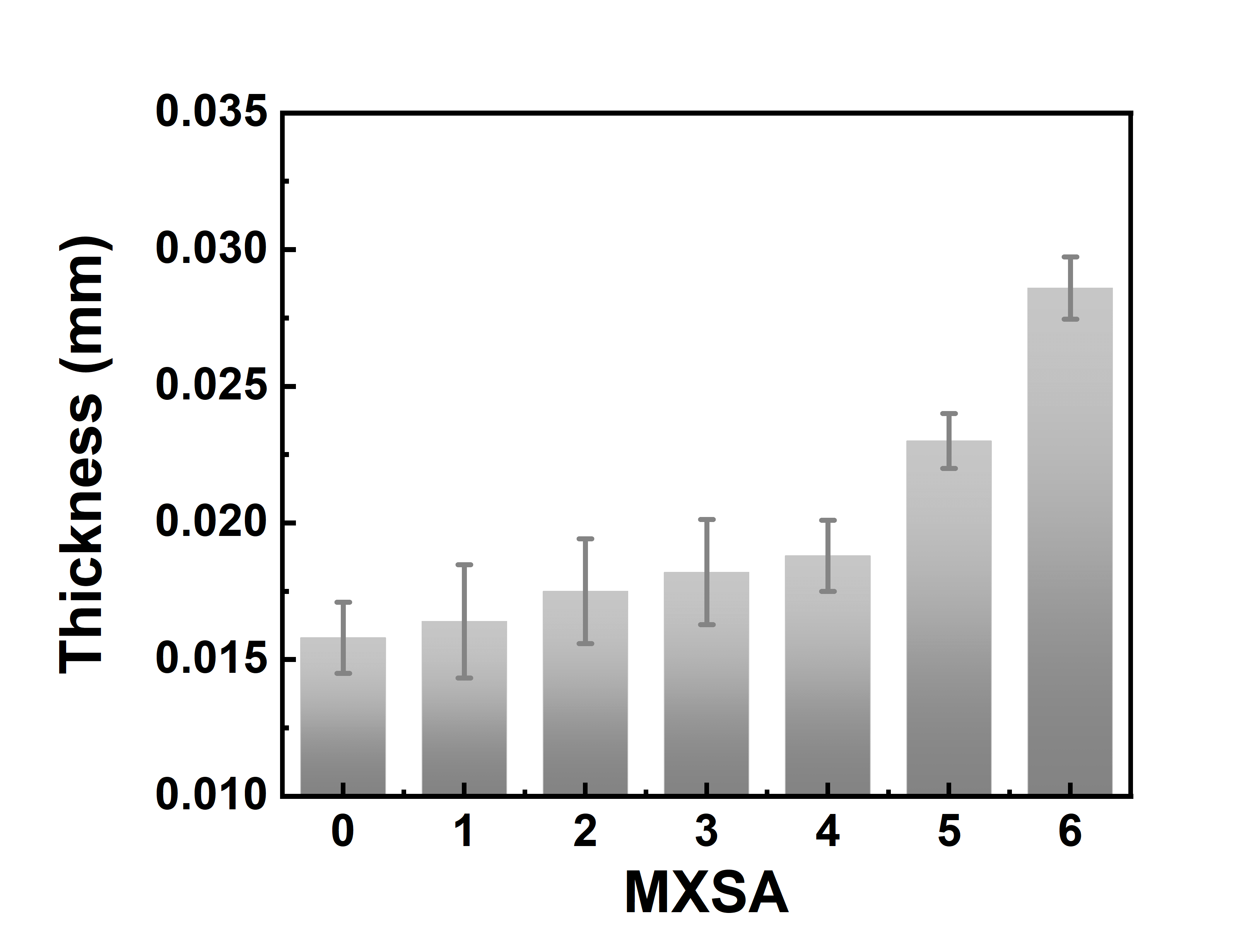


Figure S13 Thickness of MXSA composite films with different mass ratios of MXene nanosheets. Error bars represent standard deviation, n = 3.

The proportion of SA and MXene nanosheets plays a pivotal role in determining the thickness of the resultant MXSA composite films, which is also crucial for the mechanical and actuating properties of MXSA films. To guarantee the robustness and homogeneity of MXSA composite films, the initial mixture composed of 2.5 g of 2%wt SA solution and MXene suspension of different masses was made to prepare the MXSA films with various proportions. The correlation between the thickness and composition proportion of MXSA film is illustrated in Figure S13. Obviously, the result reveals a proportional increase in the thickness of MXSA composite films with the weight of the MXene suspension.


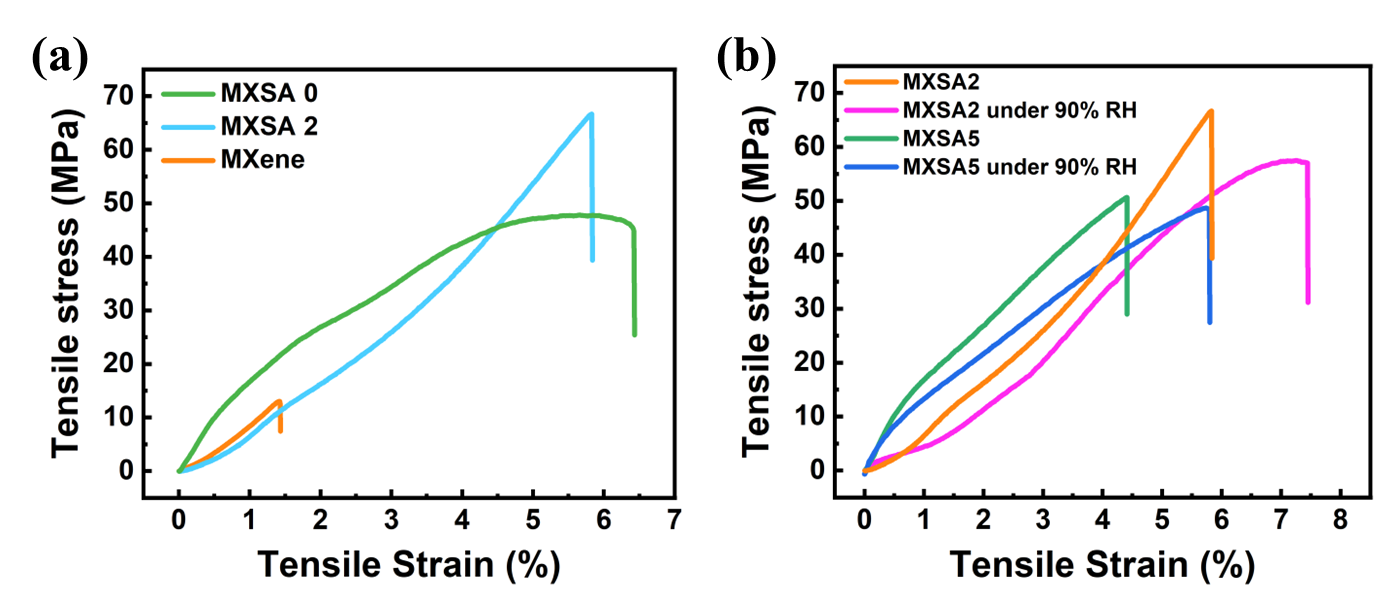


**Figure S14** (a) Tensile stress-stain curve of MXSA films and the MXene film. (b) Tensile stress-stain curve of MXSA films under 90% RH stimulation.

**
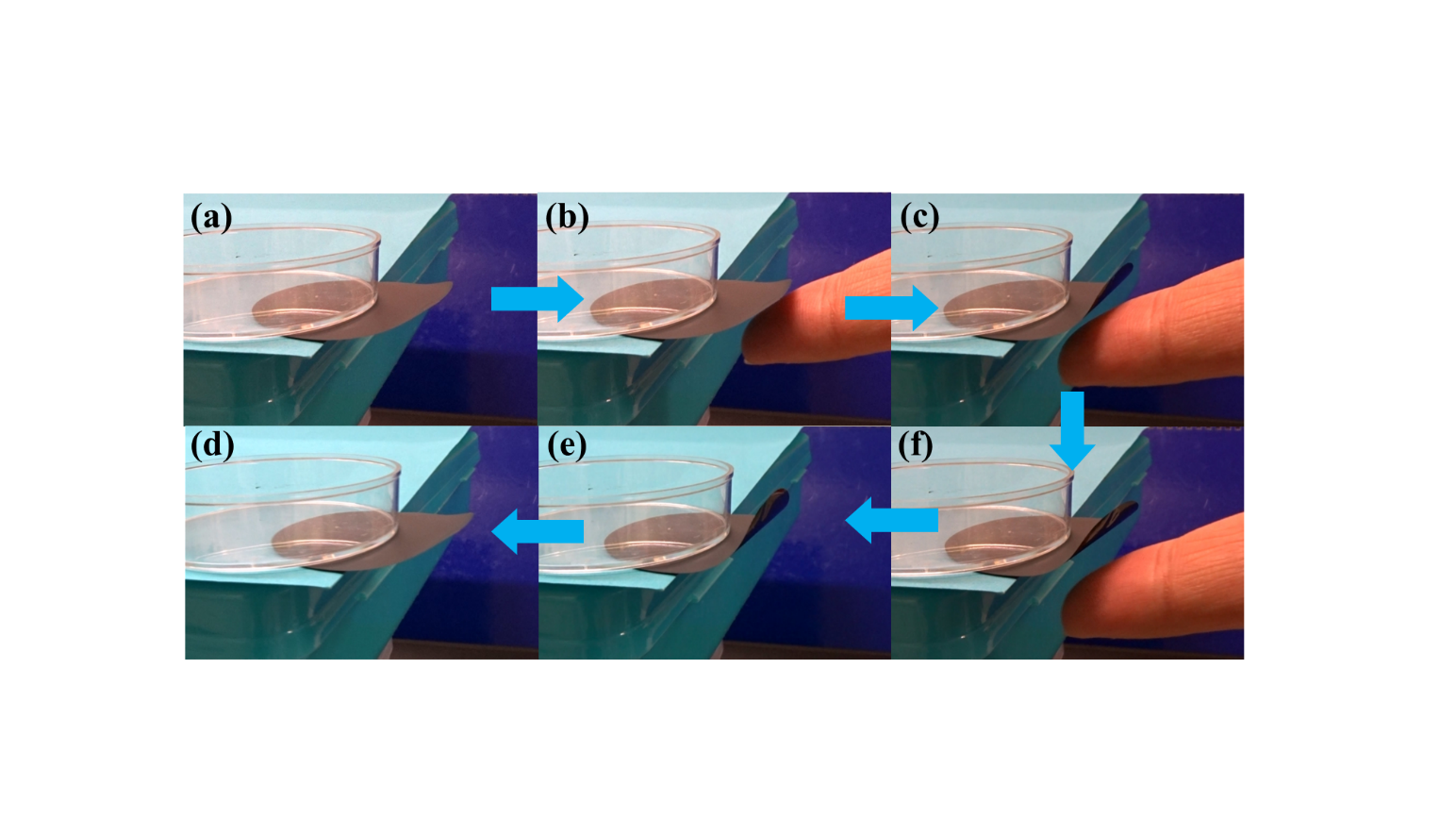
**

Figure S15 Snapshots of bending behaviour of MXSA composite actuator triggered by fingertip.

**
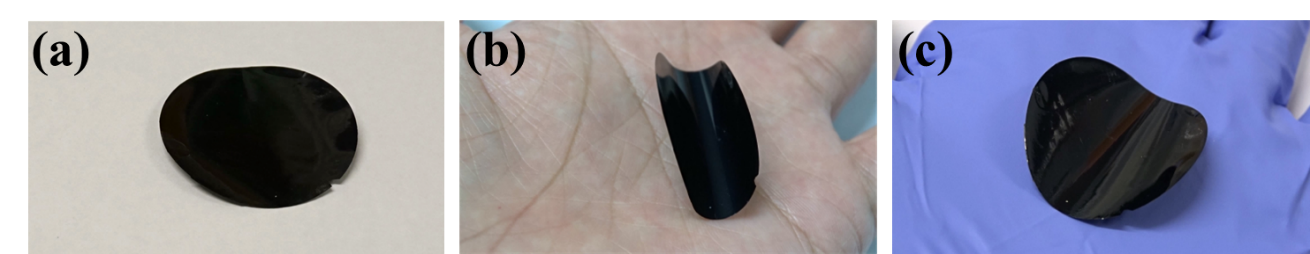
**

**Figure S16** (a) Initial flat state of the MXSA film under ambient conditions. (b) Bending behaviour of MXSA film when placed on the palm without gloves. (c) Reduced bending level of the MXSA film when placed on the palm with gloves.


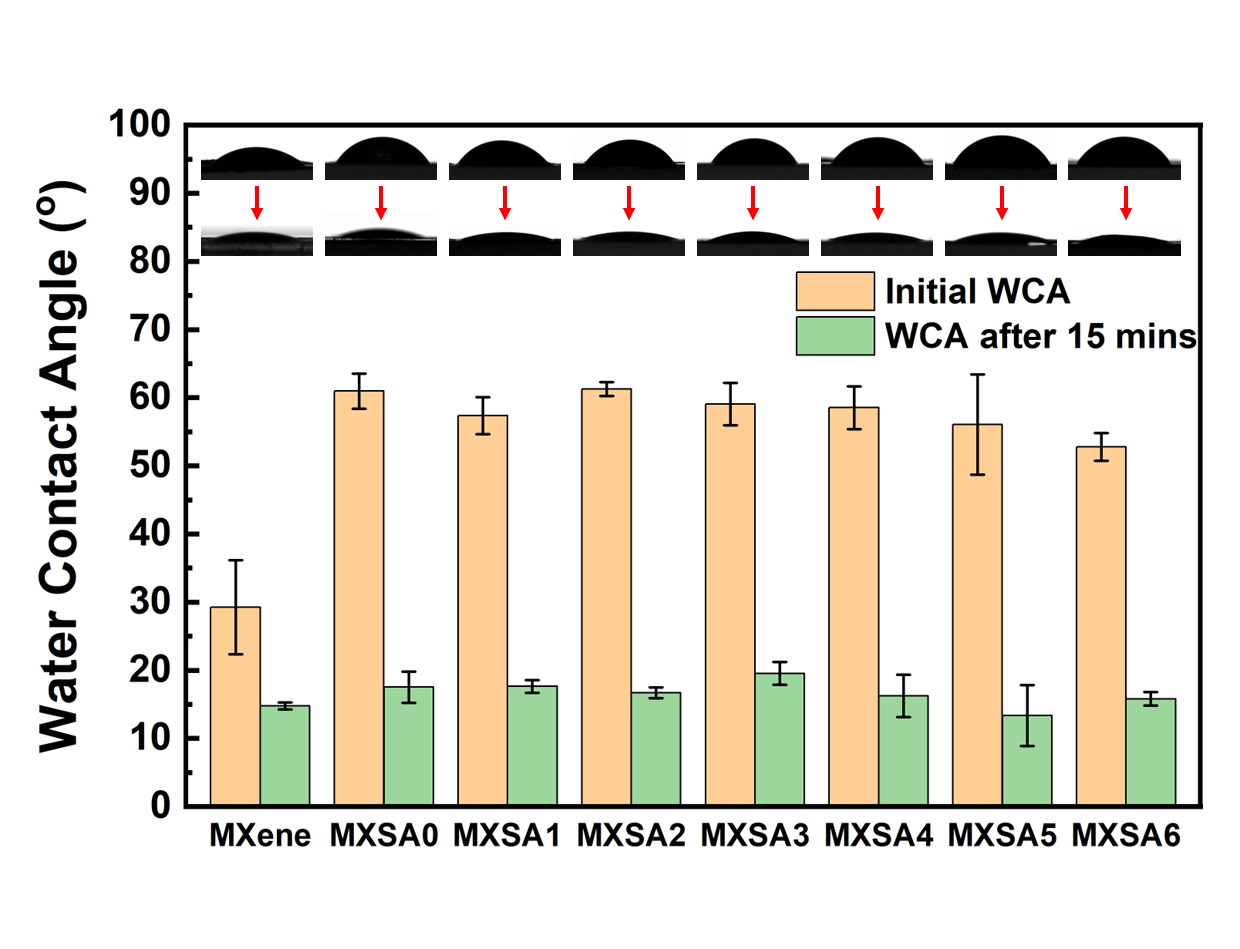


**Figure S17** Water contact angle of MXene film and MXSA composite films.


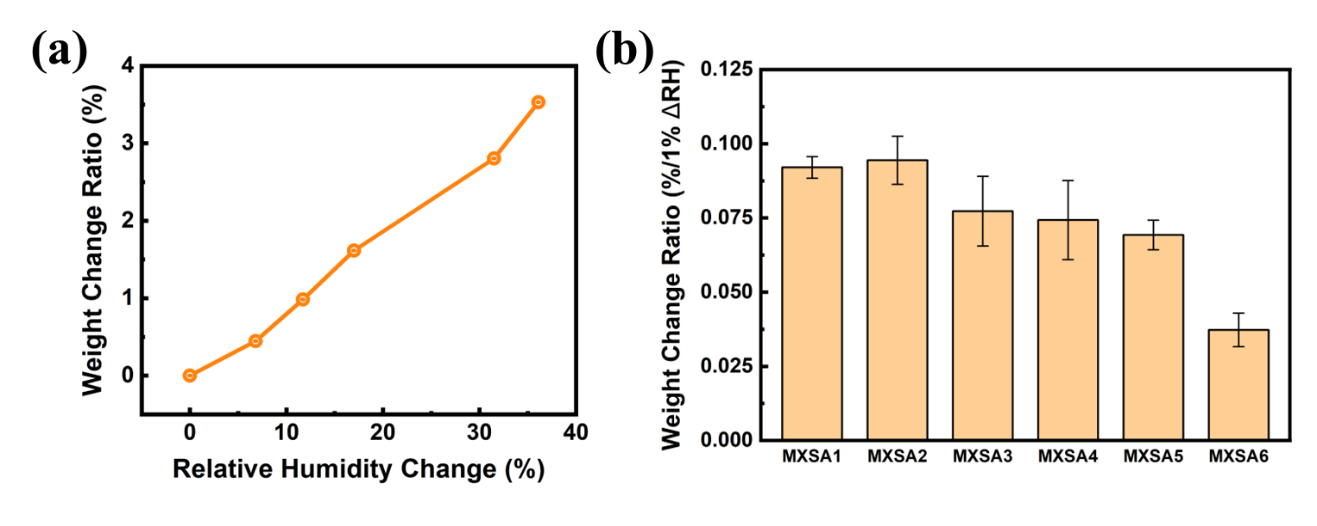


**Figure S18** (a) Weight change ratio of MXSA2 composite film as a function of relative humidity change. (b) Average weight change ratio of MXSA composite films per 1% RH change.

**
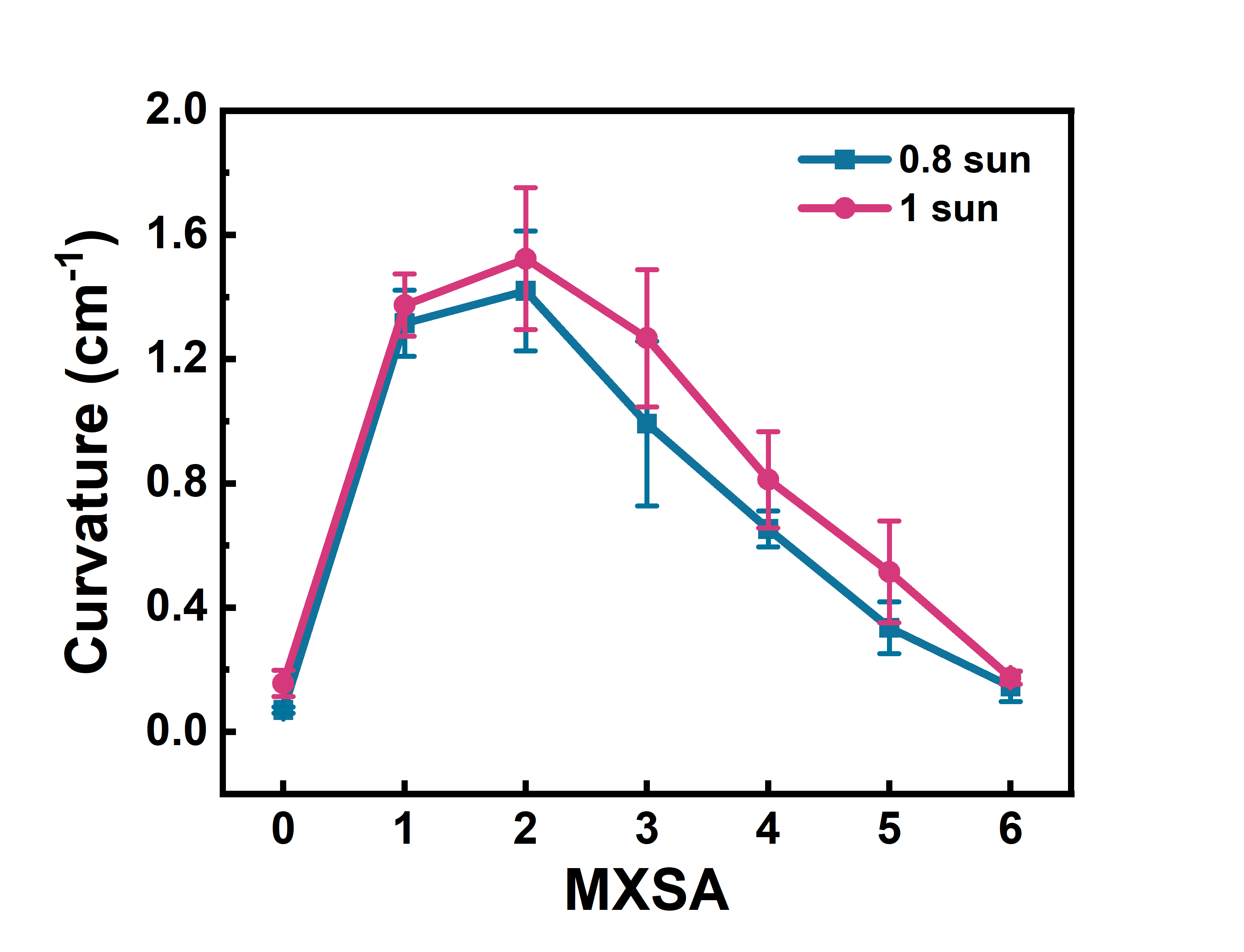
**

Figure S19 Snapshots of bending behaviour of MXSA composite actuator triggered by fingertip.


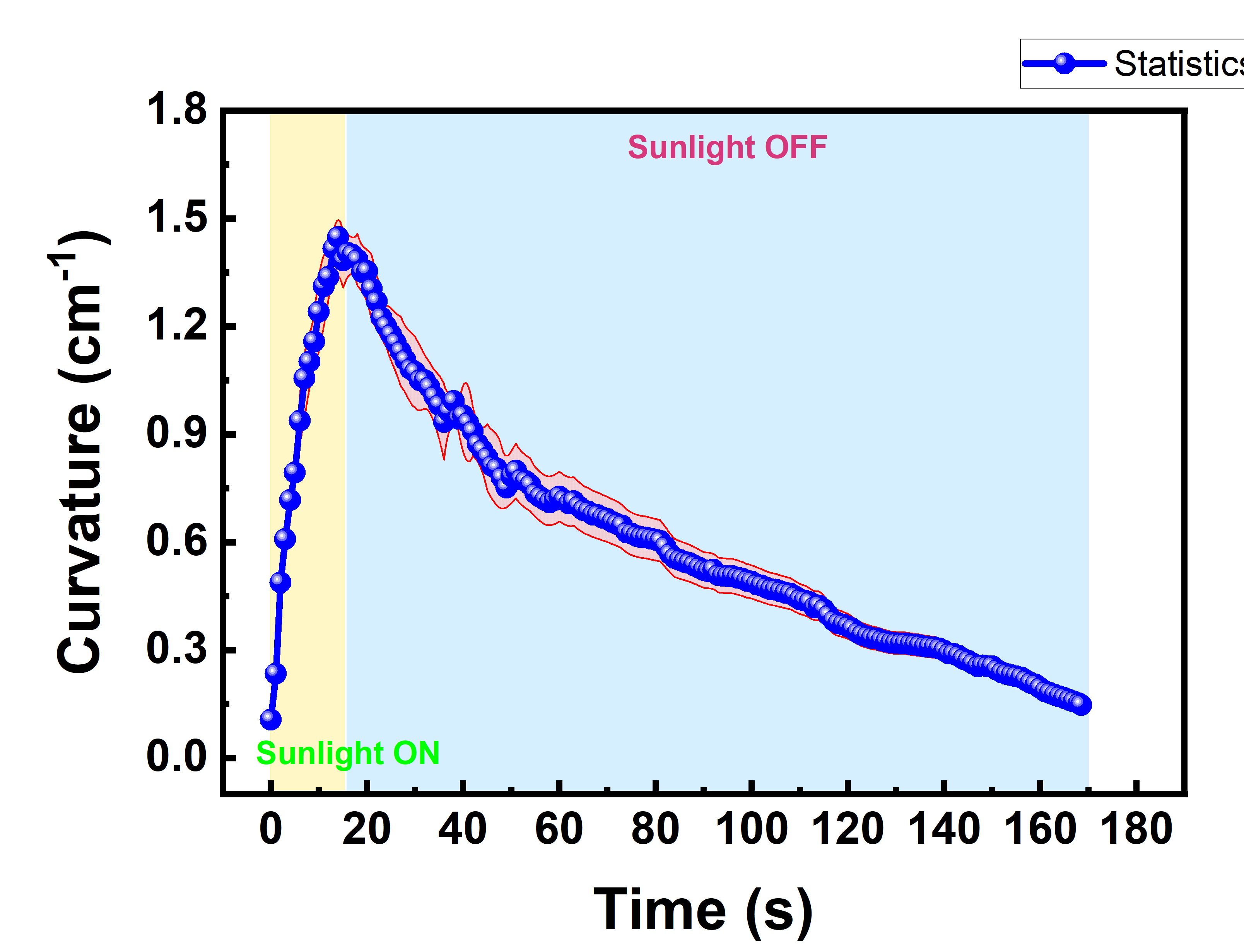


Figure S20 Real-time curvature changes of MXSA actuator under sunlight with an intensity of 1 sun. Error bars represent standard deviation, n = 3.

**Finite element analysis**

Theoretical analysis was employed to study the bending-dominated deformation of MXSA composite actuators driven by moisture and sunlight. For moisture-driven bending deformation, the curvature *k* of the actuator can be expressed by a bi-layer beam model as^[9]^

| $\kappa=\frac{6\beta\delta\left(\alpha-\alpha^{2}\right)}{h}$ | (S1) |
| --- | --- |

in which *b*, *d*, *h* and *a* denote the coefficient of moisture expansion, the change of external humidity level, the thickness of the actuator and the thickness ratio between the layer with humidity change and the whole actuator, respectively. The above equation indicates that the larger the coefficient of moisture expansion, the higher the humidity level change, the thinner the thickness of the actuator, and the greater the curvature.

Regarding the deformation caused by sunlight irradiation, the final configuration will be affected by the following two factors. One is the thermal expansion deformation caused by the increase in surface temperature of the actuator, and the other is the dehydration shrinkage deformation of the actuator due to the decrease in environmental humidity. A simple theoretical analysis is conducted on the above process. Firstly, the thermal expansion deformation can be defined as *a*(*T*_1_-*T*_0_), in which *a* is the coefficient of thermal expansion, *T*_0_ and *T*_1_ represent the initial and final temperature, respectively. Secondly, the Fick’s law is implemented here to explain the shrinkage deformation caused by humidity change. The moisture content of the actuator *M* at different time *t* after the change in environment humidity can be estimated as^[10]^

| $M=\left(1-\exp\left[-7.3\left(\frac{Dt}{4h^{2}}\right)^{0.75}\right]\right)\left(M_{m}-M_{i}\right)+M_{i}$ | (S2) |
| --- | --- |

in which *M_i_* and *M_m_* denote the initial moisture content and maximum moisture content under given environmental condition, *D* and *h* are the diffusivity and the thickness of the actuator, respectively. The above formula indicates that as the environmental humidity decreases, the moisture content of the actuator also decreases, resulting in shrinkage deformation. Considering the change of the surface temperature *T* of the actuator, the Arrhenius equation^[11]^, *D = A*e^(-^*^E^*/*^RT^*), is introduced here to represent the change in diffusion coefficient with temperature, in which *A*, *E* and *R* denote Arrhenius pre-factor, the activation energy and the gas constant, respectively. The above equation means that as the temperature increases, the diffusion coefficient of the actuator increases. The decrease in environmental humidity and the increase in diffusion coefficient of the actuator both accelerate the process of dehydration shrinkage, resulting in a more severe and dominant dehydration shrinkage deformation compared to thermal expansion deformation. Therefore, under the sunlight irradiation, the actuator undergoes dehydration shrinkage deformation, which is consistent with the experiment.

Finite element analysis (FEA) was performed with the commercial package ABAQUS^[12]^ to study the bending deformation of the actuator subjected to the external moisture and sunlight stimulation. Due to the difficulty in determining the moisture expansion coefficient and sunlight shrinkage coefficient of the actuator through experiment, the above coefficients were determined by comparing the bending angle and curvature of the actuator in experiment and FEA results. The obtained coefficient was applied to simulate the humidity-driven bending deformation of petal-shaped films, and the consistency between the simulation and the experiment verified the accuracy of the estimated coefficient.

Considering the similar expansion mechanism between the moisture and the temperature, the humidity-response performance of MXSA actuators was simulated by thermal expansion subroutines^[13]^. Besides, the sunlight illumination was simplified as a stimulus that only causes temperature changes, and an equivalent shrinkage coefficient was utilized to characterize the sunlight-driven deformation. Considering the influence of different MXene contents on the deformation of the composite films, MXSA 2 and MXSA 6 are selected for analysis. The material properties and thickness of MXSA 2 are $E_{\text{MXSA2}}=1864 \text{MPa}$, $v_{\text{MXSA2}}=0.4$ and $h_{\text{MXSA2}}=17.5\text{ μm}$, while the material properties and thickness of MXSA 6 are $E_{\text{MXSA6}}=1537 \text{MPa}$, $v_{\text{MXSA6}}=0.4$ and $h_{\text{MXSA6}}=28.6\text{ μm}$. In this simulation, the C3D8R element was employed and the hourglass control was introduced to prevent the excessive deformation of the elements. There are at least three elements distributed in the thickness direction to avoid the grid dependency.

Figure S21 shows the bending deformation of quadrilateral MXSA 2 and MXSA 6 actuators with length of 32 mm and width of 6 mm obtained by FEA under the moisture stimulation. The initial relative humidity (RH) of both types of actuators is 41%. Considering the humidity diffusion and the gap between the nozzle and the actuator, a Gaussian distribution^[14]^, with 95% confidence interval corresponding to an equivalent length of 6 mm, is adopted to represent the humidity distribution on the top surface of the actuator as

| $RH\left(x,y\right)=(RH_{\max}-RH_{0})e^{-\frac{2\left(x^{2}+y^{2}\right)}{9}}+RH_{0}$ | (S3) |
| --- | --- |

in which *RH*_max_ and *RH*_0_ denote the maximum RH and the initial RH, *x* and *y* are the coordinate of the actuator, respectively. *RH*_max_ is 91% measured by experiment in the simulation. The top end of the actuator is fixed during the simulation to avoid the rigid body displacement. As shown in Figure S21, the humidification area of the actuator undergoes significant bending deformation, with the maximum displacement occurring at the bottom, which is consistent with the experiment observation. By compared the bending angle after five different humidification conditions (*RH*_max_ are 47%, 56%, 65%, 76%, 85% and 91%, respectively), the moisture expansion coefficients of MXSA 2 and MXSA 6 are approximated as 4.8% and 3.4%, respectively.


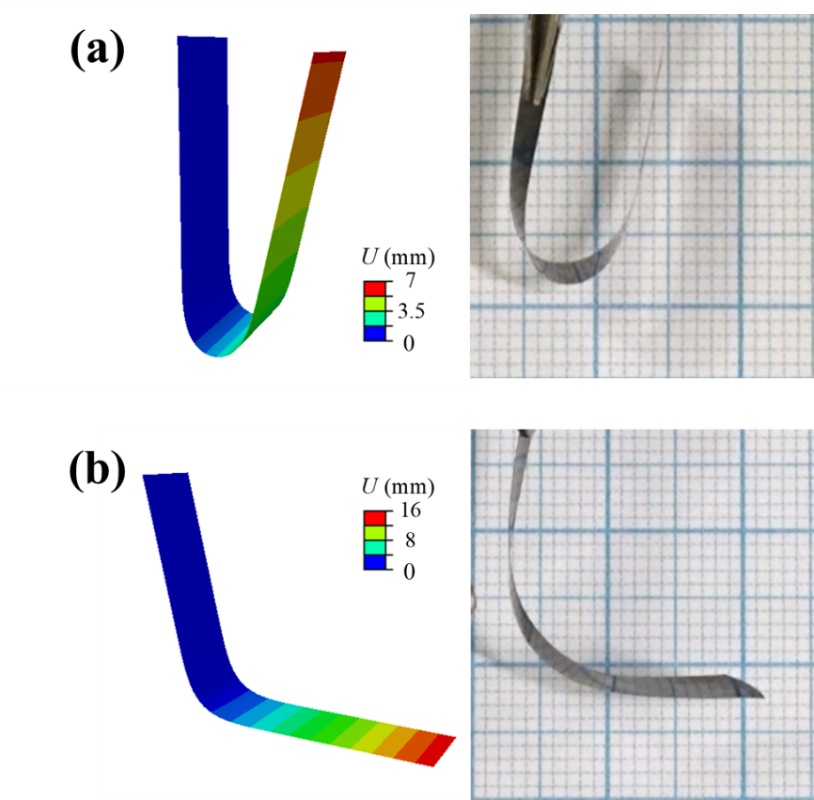


**Figure S21** Bending of quadrilateral (a) MXSA 2 and (b) MXSA 6 actuators obtained by FEA results and experiment under moisture stimuli.

Figure S22 shows the deformation of circular MXSA 2 actuator and MXSA 5 actuator with a radius of 22.5 mm simulated by FEA under sunlight exposure. The initial temperature field of both actuators is 23.5 °C. Since the sunlight intensity decays exponentially with increasing transmission distance^[15]^, an exponential function is employed to describe the temperature distribution of the actuators as

| $T_{x}=T_{\max}e^{-k\left(R-\left\vert x\right\vert\right)}$ | (S4) |
| --- | --- |

in which *T*_max_, *k* and *R* denote the maximum temperature under sunlight exposure and the exponential decay coefficient to be determined, and the radius of the actuator, respectively. *k* can be determined by substituting the temperatures from both sides and the centre of the actuator into Equation S4. It is worth noting that, the temperature of two actuators under the same sunlight exposure is different due to the different contents of MXene. MXSA 6 actuator occurs negligible temperature change and bending deformation, therefore, MXSA 5 actuator is used for the sunlight analysis in the manuscript. The material properties and thickness of MXSA 5 are $E_{\text{MXSA5}}=1450 \text{MPa}$, $v_{\text{MXSA5}}=0.4$ and $h_{\text{MXSA5}}=23\text{ }\text{μ}\text{m}$. For MXSA 2, the temperature on both sides is 50.4 °C and the temperature in the middle is 39.6 °C, while for MXSA 5 is 52.5 °C and 37.9 °C, respectively. Considering the deformation characteristic of the actuator after sunlight illumination, temperature fields are applied to both the upper and lower surfaces to make the temperature difference on both sides of the actuator equal to zero, while the temperature difference in the middle is equal to the temperature change of the lowest point after bending deformation. In the simulation, the middle area with width of 0.1 mm is fixed to avoid rigid body displacement. The FEA result is consistent with the experimental observation that the middle area occurs the significant bending deformation due to the large temperature difference and both sides of the actuator have the maximum displacement, as shown in Figure S22. The equivalent shrinkage coefficients of MXSA 2 and MXSA 5 are approximately determined to be 0.02% and 0.009%, respectively, by comparing the curvature under four types of sunlight exposure (20 mw/cm^-2^, 40 mw/cm^-2^, 60 mw/cm^-2^, 80 mw/cm^-2^ and 100 mw/cm^-2^) obtained by FEA results and experiment.


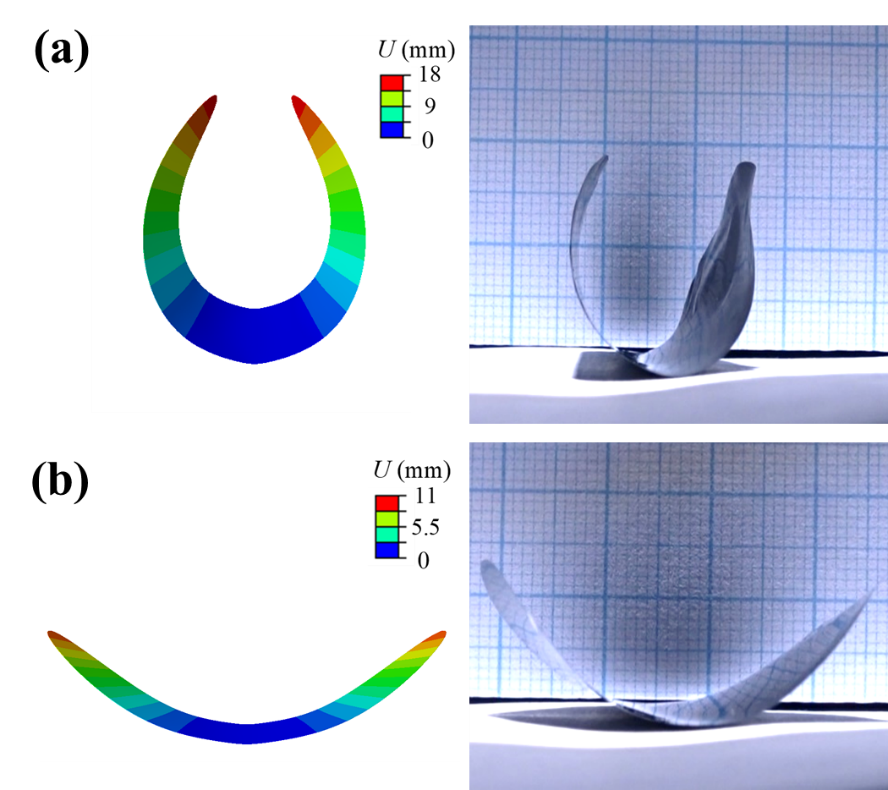


**Figure S22** Bending of circular (a) MXSA 2 and (b) MXSA 5 actuators obtained by FEA results and experiment under sunlight exposure.

Finally, the deformation of a flower-inspired actuator driven by humidity was simulated, as shown in Figure S23. The flower-inspired actuator includes three parts, the upper petals, the lower petals and the middle connecting area. The upper petals are composed of four equilateral-triangle-shape MXSA 2 films with the side length of 15 mm and spacing angle of 30°. The lower petals are composed of four fan-shape MXSA 6 films with radius of 22 mm, centre angle of 60°, and spacing angle of 30°. The middle connecting area is a circle with radius of 2.5 mm and has no deformation during humidification, which is modelled as a rigid body. The initial humidity is 60% and *RH*_max_ is 90%. Considering the different humidification methods compared to quadrilateral actuators and the fact that the lower petal has a certain blocking effect on the upper petal, the equivalent length of 95% confidence interval in the Gaussian distribution are set as 12.8 mm and 18.6 m for the upper and lower layers, respectively. FEA results shows that the flower-inspired actuator bend upwards during humidification, similar to the effect of petal closure in nature. Besides, significant bending deformation occurs in the humidification area, except for the middle connecting area, and the bending deformation of the upper petals is greater than that of the lower petals due to the difference in moisture expansion coefficient, which is consistent with experimental observation.


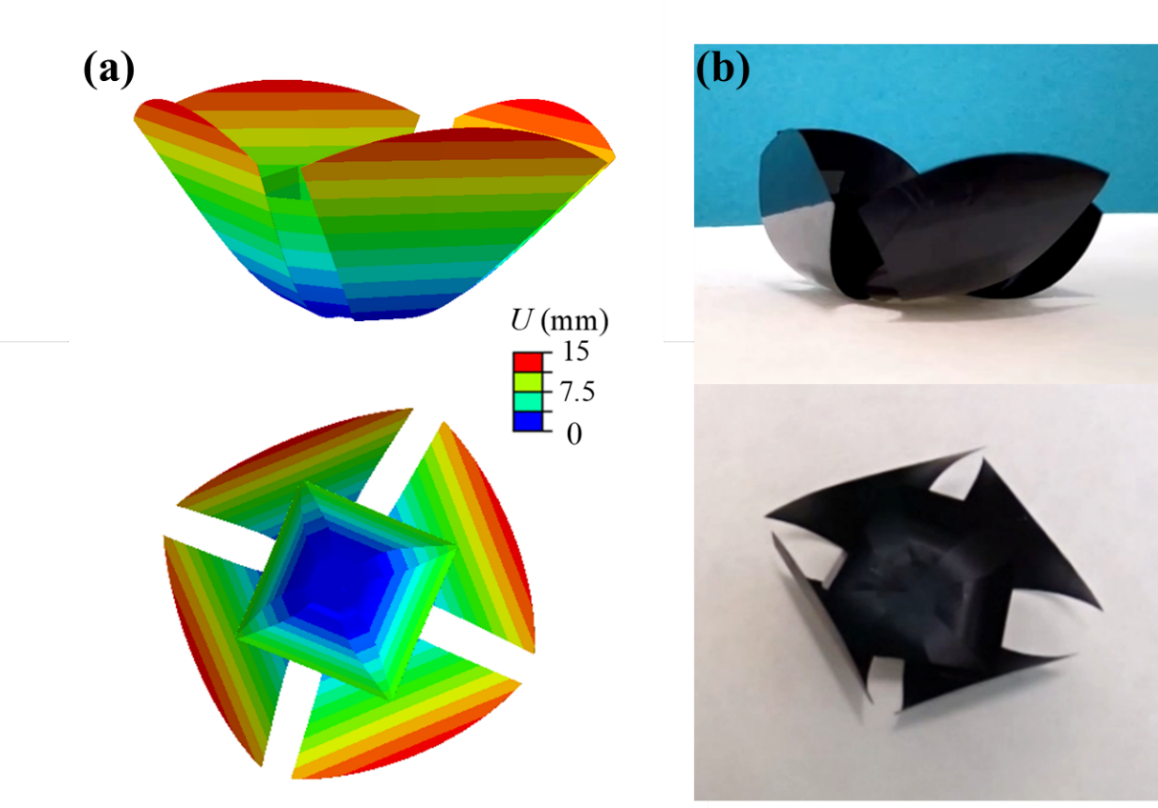


**Figure S23** Bending of flower-inspired actuator in (a) FEA and (b) experiment.


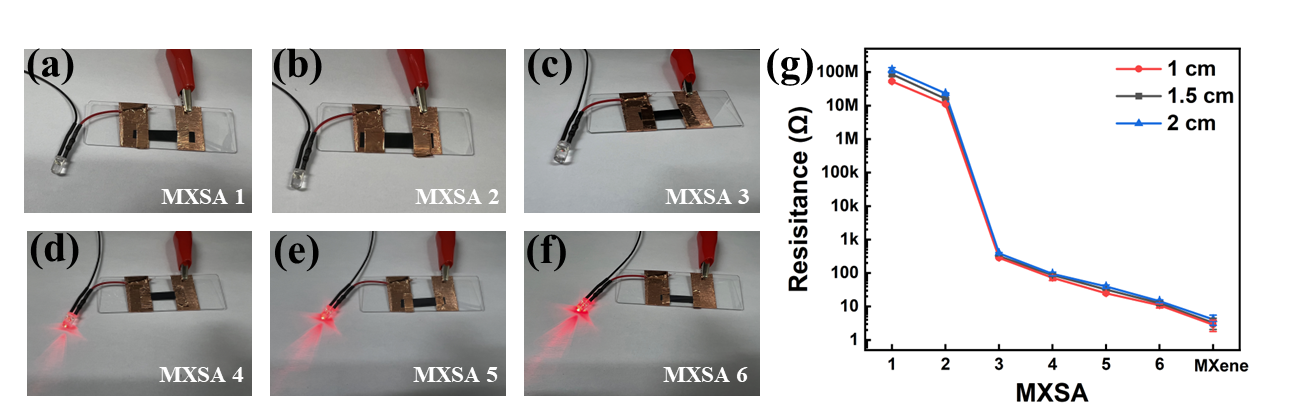


Figure S24 (a-f) Photographs of the closed circuits using MXSA film strips as conductors (g) Surface resistance of MXSA composite films measured at different distances and with varying mass ratios of MXene nanosheets.

Table S1 Comparison of MXSA based actuator with other actuating materials

| **Actuating materials** | **Stimuli** | **Bending angle/curvature** | **Response time** | **Tensile strength** | **Ref.** |
| --- | --- | --- | --- | --- | --- |
| GO/PPy | NIR light/Humidity | 1.4 cm^–1^/4.18 cm^−1^ | 3 s/9 s | 700 kPa | [16] |
| Graphene-conjugated PEDOT:PSS | Electricity | 2.15 cm^–1^ | 300 s | 35 MPa | [17] |
| MXC/PDMS | NIR light/ Electricity | 219°/209° | 3.5 s/20 s | - | [18] |
| MXene/PEDOT:PSS-Integrated PNIPAM Hydrogels | NIR light | 50° | 7 s | 140 kPa | [19] |
| LM@A-MXene PNIPAM hydrogels | NIR light | 55° | - | 10 kPa | [20] |
| GO/Bacterial cellulose | Humidity/NIR light/Electricity | 148°/134° | 60 s/10 s/10 s | 51.86 MPa | [21] |
| GO/Fe_3_O_4_/Starch | Humidity | 180° | 2.5 s | 78 MPa | [22] |
| Polyaniline/RGO/CNT | NIR | 0.86 cm^-1^ | 10 s | - | [23] |
| MXSA | Humidity/Sunlight | 190°/1.45 cm^-1^ | 14 s | 70 MPa | This work |

**Reference:**

[1] M. Mahmood, A. Rasheed, I. Ayman, T. Rasheed, S. Munir, S. Ajmal, P. O. Agboola, M. F. Warsi, M. Shahid, *Energy & Fuels* **2021**, 35, 3469.

[2] D. Liu, T. Li, W. Sun, W. Zhou, G. Zhang, *ACS Omega* **2022**, 7, 31945.

[3] N. U. Kiran, A. B. Deore, M. A. More, D. J. Late, C. S. Rout, P. Mane, B. Chakraborty, L. Besra, S. Chatterjee, *ACS Appl. Electron. Mater.* **2022**, 4, 2656.

[4] S. Nam, S. Umrao, S. Oh, K. H. Shin, H. S. Park, I.-K. Oh, *Compos. B Eng.* **2020**, 181, 107583.

[5] A. Iqbal, N. M. Hamdan, *Materials* **2021**, 14, 6292.

[6] a) A. Sengupta, B. B. Rao, N. Sharma, S. Parmar, V. Chavan, S. K. Singh, S. Kale, S. Ogale, *Nanoscale* **2020**, 12, 8466; b) M. Xu, L. Li, W. Zhang, Z. Ren, J. Liu, C. Qiu, L. Chang, Y. Hu, Y. Wu, *Macromol. Mater. Eng.* **2023**, 308, 2300200.

[7] F. Liu, A. Zhou, J. Chen, H. Zhang, J. Cao, L. Wang, Q. Hu, *Adsorption* **2016**, 22, 915.

[8] M. Han, X. Yin, H. Wu, Z. Hou, C. Song, X. Li, L. Zhang, L. Cheng, *ACS Appl. Mater. Interfaces* **2016**, 8, 21011.

[9] a) L. Sun, L. Che, M. Li, W. G. Neal, X. Leng, Y. Long, Y. Jia, Y. Gao, M. Palma, Y. Lu, *SusMat* **2023**, 3, 207; b) S. Timoshenko, *J. Opt. Soc. Am.* **1925**, 11, 233.

[10] C.-H. Shen, G. S. Springer, *Journal of Composite Materials* **1976**, 10, 2.

[11] I. Matsumoto, K. Asano, K. Sakaki, Y. Nakamura, *Int. J. Hydrogen Energy* **2011**, 36, 14488.

[12] D. Systèmes, *Simulia Corp. Providence, RI, USA* **2007**, 40.

[13] Y. Lv, Q. Li, J. Shi, Z. Qin, Q. Lei, B. Zhao, L. Zhu, K. Pan, *ACS Appl. Mater. Interfaces* **2022**, 14, 12434.

[14] L. Salkin, A. Schmit, P. Panizza, L. Courbin, *Physical Review Letters* **2016**, 116, 077801.

[15] Beer, *Annalen der Physik* **1852**, 162, 78.

[16] Y. Dong, J. Wang, X. Guo, S. Yang, M. O. Ozen, P. Chen, X. Liu, W. Du, F. Xiao, U. Demirci, *Nat. Commun.* **2019**, 10, 4087.

[17] W. Liu, Z. Lei, W. Xing, J. Xiong, Y. Zhang, P. Tao, W. Shang, B. Fu, C. Song, T. Deng, *ACS Nano* **2023**, 17, 16123.

[18] H. Li, Z. Wu, Y. Xing, B. Li, L. Liu, *Nano Energy* **2022**, 103, 107821.

[19] P. Xue, C. Valenzuela, S. Ma, X. Zhang, J. Ma, Y. Chen, X. Xu, L. Wang, *Adv. Funct. Mater.* **2023**, 2214867.

[20] S. Ma, P. Xue, C. Valenzuela, X. Zhang, Y. Chen, Y. Liu, L. Yang, X. Xu, L. Wang, *Adv. Funct. Mater.* **2024**, 34, 2309899.

[21] K. Yang, W. Cai, M. Lan, Y. Ye, Z. Tang, Q. Guo, M. Weng, *Soft Matter* **2022**, 18, 9057.

[22] H. Chathuranga, I. Marriam, S. Chen, Z. Y. Zhang, J. MacLeod, Y. N. Liu, H. Yang, C. Yan, *ACS Appl. Mater. Interfaces* **2022**, 14, 16772.

[23] Q. Guo, C. Li, K. Yang, P. Zhou, N. Hua, M. Weng, *ACS Appl. Nano Mater.* **2023**, 6, 4925.
